# Insights Into Overall Photocatalytic Water Splitting Through Simultaneous In Situ H_2_ and O_2_ Measurements

**DOI:** 10.1002/cssc.202502721

**Published:** 2026-03-08

**Authors:** Nadzeya Brezhneva, Alexander Eith, Ebrahim Abedini, Daniel Kowalczyk, Dirk Ziegenbalg, Jacob Schneidewind

**Affiliations:** ^1^ Institute of Organic Chemistry and Macromolecular Chemistry Friedrich Schiller University Jena Jena Germany; ^2^ Center for Energy and Environmental Chemistry Jena (CEEC Jena) Friedrich Schiller University Jena Jena Germany; ^3^ Institute of Chemical Engineering Ulm University Ulm Germany; ^4^ Jena Center for Soft Matter (JCSM) Friedrich Schiller University Jena Jena Germany; ^5^ Helmholtz Institute for Polymers in Energy Applications Jena (HIPOLE Jena) Jena Germany

**Keywords:** hydrogen, in situ measurements, kinetics, photocatalysis, photoreactor, water splitting

## Abstract

Photocatalytic overall water splitting is a promising pathway to produce green hydrogen but also presents unique research challenges due to the need to detect both gaseous products (H_2_ and O_2_). While gas chromatography (GC) is the most commonly employed method in this context, it faces multiple shortcomings: low time resolution as well as the need to alter the reaction conditions (vacuum or carrier gas flushing) to feed the products into the GC, which limits the extent to which obtained insights can be translated into scalable photoreactors (where H_2_ and O_2_ accumulate). Against this backdrop, we report a novel, sensor‐based experimental method which allows for simultaneous in situ detection of H_2_ and O_2_ in both the liquid and gas phase. It is based on a standardized modular photoreactor platform and integrates optical O_2_ and electrochemical H_2_ sensors for real‐time measurements during water splitting. Using this method, we investigate photocatalytic overall water splitting using Rh_2−*y*_Cr_*y*_O_3_/Al:SrTiO_3_, determining the irradiance dependence, thermal activation barrier, optimal cocatalyst loading, and H/D kinetic isotope effect. This highlights the versatility of the described method as well as the depth of information that can be obtained through the in situ H_2_/O_2_ detection approach.

## Graphical abstract

A photoreactor setup allowing for simultaneous *in situ* detection of H_2_ and O_2_ in the liquid as well as gas phase is described. Using this system, overall water splitting with Rh_2−*y*_Cr_*y*_O_3_/Al:SrTiO_3_ is investigated, determining irradiance dependence, thermal activation barrier, optimal cocatalyst loading, and H/D kinetic isotope effect.

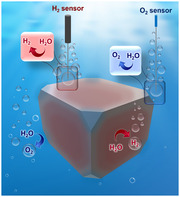

## 1 Introduction

Photocatalytic overall water splitting, wherein water is split into hydrogen and oxygen, offers a promising route for the scalable conversion of sunlight to green hydrogen [1, 2, 3]. Various photocatalysts have been developed for this reaction, largely based on semiconductors such as TiO_2_ [4], SrTiO_3_ [5], C_3_N_4_ [6], and others [7]. A crucial aspect of photocatalytic overall water splitting research, and thus a prerequisite for further progress in the field, is the reliable detection of the two reaction products, H_2_ and O_2_. Aside from indicating the catalyst's activity, quantification of both gases gives insights into whether the reaction really does occur stoichiometrically (forming the expected 2:1 H_2_/O_2_ ratio) and can give insights into the reaction kinetics [8, 9]. The vast majority of published works employ gas chromatography (GC) to quantify H_2_ and O_2_ in the gas phase, often in a closed gas circulation system allowing for online measurements [8, 9].

While this approach has been widely employed, it also presents multiple challenges: (1) The entire photocatalytic setup is complex and relatively expensive (especially due to the GC component), creating challenges for accessibility and high‐throughput work. (2) The use of a GC typically requires either flushing the reaction solution with an inert carrier gas or operating it under vacuum to feed the product gases into the system. These interventions often positively impact photocatalytic performance, since overall water splitting systems generally show higher activity under reduced background pressure and in the absence of H_2_/O_2_ in the gas phase [10]. However, those conditions are not representative of scalable photoreactor systems, where H_2_ and O_2_ accumulate in the gas phase [3]. (3) The time resolution of GC is limited, usually on the scale of minutes. This low time resolution limits the kinetic insights that can be gained, since the long measurement time scales conflate intrinsic kinetic effects (e.g., temperature dependence) with slower processes (e.g., catalyst degradation) [11]. This is compounded by the fact that gas phase measurements cannot provide insights into the initial reaction phase, since H_2_ and O_2_ are initially formed in the liquid phase and must diffuse into the gas phase before they can be detected. Alternative methods to GC have been reported in the literature, such as electrochemical or optical sensing of O_2_ [12, 13, 14], and in the context of photoelectrochemical water splitting, H_2_ has also been detected in situ using electrochemical microsensors [15, 16]. Electrochemical O_2_ sensors have also been used to study immobilized photocatalyst films [17], and photoscanning electrochemical microscopy has been applied to investigate H_2_ and O_2_ formation by single photocatalyst particles [18, 19]. However, such approaches have, to the best of our knowledge, not been applied in overall photocatalytic water splitting using suspended photocatalysts, which is the most common way that photocatalysts are investigated in the literature.

A second challenge for photocatalysis research is the reproducibility of experiments, since photocatalytic reactions are highly sensitive to the precise irradiation parameters [8]. Progress has been made on the development of standardized modular photoreactor platforms [20], but there is still a strong need for reproducible experimental setups to facilitate comparisons between photocatalysts and enable structured progress in the field [21, 22].

Against this backdrop, we are introducing a novel photoreactor setup based on a standardized modular reactor platform that integrates electrochemical/optical sensors to enable simultaneous in situ detection of H_2_ and O_2_ in both the gas and liquid phases. This low‐cost, fully open‐source, documented, and reproducible system allows for precise control of all relevant reaction conditions (irradiance, temperature, gas phase composition) and does not require any carrier gases or changes to the reaction conditions. It therefore allows for the collection of photocatalytic performance data under true *operando* conditions that are directly relevant to scalable photoreactor systems. Importantly, the highly time‐resolved measurements (on the level of seconds) of H_2_ and O_2_ enabled by the used sensors give rich kinetic insights in both the gas and liquid phases. This provides information on the initial reaction phase, which allows for precise determination of the kinetic influence of reaction parameters (irradiance, temperature, etc.) without conflating them with slower processes such as catalyst degradation. We use this setup to investigate overall water splitting using the previously reported Rh_2*−y*_Cr_*y*_O_3_/Al:SrTiO_3_ photocatalyst [23, 24, 25], studying the influence of temperature, irradiance, and cocatalyst loading on the initial reaction rate in the liquid phase and measuring the H/D kinetic isotope effect (KIE). Through these investigations, we find that liquid phase measurements can reproduce previously reported reactivity trends while also providing new insights that require further investigations, such as a H/D KIE which is, surprisingly, higher than previously reported.

## 2 Results and Discussion

### 2.1 Photocatalytic Reactor Setup

**FIGURE 1:**
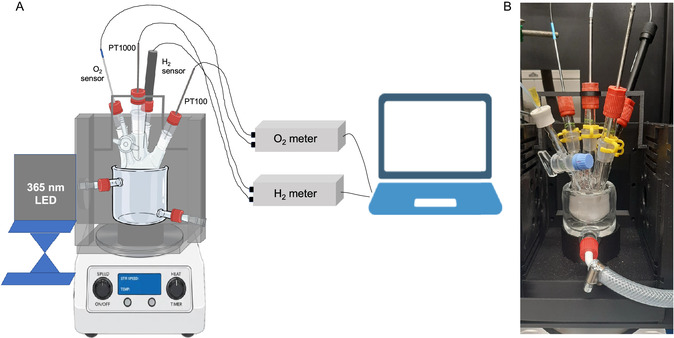
(A) Schematic overview of light source (365 nm LED), 3D‐printed irradiation chamber (black), double‐walled beaker for temperature control, glass photoreactor, and H_2_ as well as O_2_ sensors with temperature probes for compensation. (B) Photo of experimental setup.

The goal for the photocatalytic reactor setup is to offer precise control over irradiance, temperature, and the composition of the gas phase while also allowing for in situ detection of H_2_ and O_2_. To address the control of the reaction conditions and to expand on a standardized modular reaction platform, we modified the previously reported modular photoreactor design [20]. Based on this design, we created the 3D‐printed irradiation chamber (see Figure 1), which allows for precise placement of light source and reactor, keeping all distances constant so as to enable reproducible irradiation experiments (since irradiance strongly varies with distance from the light source).

The irradiation chamber also allows for the incorporation of a double‐walled beaker, which is connected to a thermostat to precisely control the reaction temperature. This is crucial to both avoid inadvertent heating of the reactor due to irradiation and to enable the reaction to be performed at different set temperatures to study the temperature dependence of the water splitting reaction. The reaction is performed in a glass Schlenk photoreactor, which has four glass connections as well as a valve. Being a Schlenk flask, the gas composition can be precisely controlled using standard Schlenk technique (e.g., applying vacuum, filling with an inert gas, or filling with a defined H_2_/O_2_ mixture). The four glass connections allow for the installation of up to four sensors for H_2_ and O_2_ detection. In this study, we have utilized an electrochemical H_2_ microsensor and an optical O_2_ sensor. As the measurement signals of both sensors are sensitive to temperature, temperature sensors are also installed in the reactor to provide real‐time temperature compensation. All sensors are connected to the respective H_2_/O_2_ meters, which read out the signal and provide the data to a computer. Importantly, the used sensors can be applied in both the gas and liquid phases (by adjusting their position within the reactor), allowing for studying product formation in both phases. The reaction solution is stirred using a magnetic stirring bar, ensuring homogeneous distribution of photocatalyst particles and avoiding local concentration differences.

Detailed information on the used components can be found in Supporting Information Section 4, and the data repository contains all necessary files for 3D printing, allowing other researchers to replicate this experimental setup.

A crucial aspect for the described method is the accuracy and reliability of the applied sensors for quantifying H_2_ and O_2_. The used sensors have specified detection limits of 0.3 μM (H_2_) and 0.1 μM (O_2_) in the liquid phase and 0.04 vol% (H_2_) and 0.005 vol% (O_2_) in the gas phase. Their upper measurements limits are 2000 μM (H_2_) and 500 μM (O_2_) (liquid phase) and 100 vol% (H_2_)/10 vol% (O_2_) (gas phase, but higher range O_2_ sensors are commercially available). These measurement ranges enable H_2_/O_2_ quantification across a wide range of photocatalyst activities and exceed the detection ranges of most GC systems (further information on the specifications of the used sensors can be found in Supporting Information Section 4.4.1). To ensure reliable quantification, we carefully calibrated the applied sensors (see Supporting Information Section 4.4.1 for full information). In the case of the H_2_ sensor, we found excellent linearity across the measurement range in both gas and liquid phases. We also performed a calibration experiment where we compared the H_2_ sensor results to those obtained via GC (quantification of H_2_ in the gas phase, see Supporting Information Section 4.4.3). For the O_2_ sensor, the optical detection method follows a well‐established Stern–Volmer quenching behavior, and hence, a two‐point calibration is sufficient. Aside from the calibration, the accuracy and reliability of the sensors can be impacted by temperature, pressure changes, stray light, or formation of gas bubbles (which can interfere with liquid phase measurements). In Supporting Information Section 4.4.2, we discuss those potential error sources in detail and how they are mitigated.

An important difference between the sensor‐based method described herein and GC‐based methods is that no carrier gas or vacuum is required to transport the formed gases to the measurement device (as is required when using GC). Instead, the measurement is performed in situ. This aspect is particularly relevant for photocatalytic water splitting, since the thermodynamics of the reaction (2 H_2_O → 2 H_2_ + O_2_) are dependent on the partial pressure of H_2_ and O_2_, following Le Chatelier's principle. In a closed system, H_2_ and O_2_ accumulate and their partial pressure increases over the course of the reaction, making the water splitting thermodynamically less favorable over time. This encourages the backward reaction (2 H_2_ + O_2_ → 2 H_2_O). Flushing the reaction system with carrier gas or applying vacuum reduces the partial pressure of both gases, making the reaction more thermodynamically favorable. This pressure dependence has been confirmed experimentally and differs between photocatalysts [10], depending on how well the catalyst structure can suppress the backward reaction.

Therefore, having to use carrier gases or vacuum with GC systems places a crucial experimental constraint on the investigation of pressure effects, which is avoided using the described sensor method. Importantly, in scalable reactor systems, such as glass panel reactors with immobilized photocatalysts [3], H_2_ and O_2_ do indeed accumulate in the gas phase, since continuous flushing with inert gas or application of vacuum is neither energy efficient nor economically viable [3]. Thus, the reaction conditions that can be used with the sensors (significant partial pressure of H_2_/O_2_) are likely more representative for scalable systems compared to conditions which employ carrier gas/vacuum.

### 2.2 Photocatalytic Reaction System

To investigate photocatalytic overall water splitting using the described reactor setup, we prepared a previously reported Rh_2*−y*_Cr_*y*_O_3_/Al:SrTiO_3_ photocatalyst [5, 23, 24], adapting a procedure from Osterloh and coworkers (see Supporting Information Section 3) [25]. This photocatalyst is composed of aluminum‐doped SrTiO_3_ particles (size range of 500–2000 nm, see Supporting Information Section 3.6), which are loaded with a mixed rhodium/chromium oxide (typically 0.1 wt% Rh/Cr). The Al:SrTiO_3_ particles serve as light absorbers, while the Rh_2*−y*_Cr_*y*_O_3_ particles serve as cocatalysts for the hydrogen evolution reaction [23]. This catalyst is known to stoichiometrically split water into H_2_ and O_2_ when irradiated with UV light (e.g. 365 nm) in an aqueous suspension [23, 25].

### 2.3 Data Processing Workflow

**FIGURE 2:**
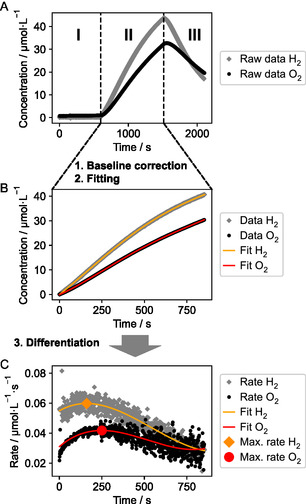
Workflow for the processing of experimentally collected H_2_/O_2_ data, illustrated for liquid phase data (processing is performed analogously for gas phase data). (A) Raw liquid phase data for H_2_/O_2_, showing the three experiment phases: (I) pre‐reaction baseline, (II) reaction phase (irradiation), and (III) postreaction phase, showing diffusion of gases into the gas phase. (B) Reaction phase data is baseline corrected and the start of irradiation is set to *t* = 0 s. Furthermore, polynomial fits to the data are shown. (C) Numerically differentiated rate data along with the differentiated polynomial fit, from which the maximum rates of H_2_ and O_2_ formation are determined. Note: the shown rate data is based on numerical differentiation of experimental data which has been smoothed using a Savitzky–Golay filter to reduce the noise level for visualization. Details on the data processing workflow can be found in Supporting Information Section 8.1.

With both the photoreactor setup and catalyst in hand, we collected the experimental data and established a corresponding data processing workflow (see Figure 2).

The collected raw data can be divided into three phases (see Figure 2A): (I) the pre‐reaction baseline before irradiation is turned on, (II) reaction phase, during which irradiation is turned on, and (III) the postreaction phase (irradiation turned back off again). For liquid phase measurements, it can be observed that the gas concentration decreases during this phase due to diffusion into the gas phase (see Figure 2A).

The reaction phase is selected for further processing, based on the start and end time of the irradiation, and baseline corrected as well as shifted on the *x*‐axis so that the start of the reaction is at *t*  = 0 s. The first 60 s after the start of the irradiation is removed in this step as this period is typically strongly affected by diffusion of gases into the sensors as well as artifacts arising from the small (<0.5°C) but sudden temperature change due to warming by the irradiation. To the reaction phase data (Figure 2B), a polynomial is fitted. This smooth polynomial allows for accurate extraction of rate data upon numerical differentiation (see Figure 2C), as the experimental data is quite noisy after differentiation.

For this study, the key kinetic information we use for the analysis of experiments is the maximum rate, which is determined by picking the maximum value of the differentiated polynomial fit (see Figure 2C). The maximum rate is used as a proxy for the initial rate, which itself cannot be determined directly due to diffusion of gases into the sensor at the beginning of the reaction, which leads to a short induction period.

However, as the full temporal evolution of concentration and rates is available, other kinetic information can be obtained from the data, for example, by fitting full kinetic models.

Considering the data in Figure 2, we can make two observations: First, the reaction rate initially increases, reaches a maximum, and then decreases. The initial increase can likely be explained by diffusion of gases within the liquid phase to reach the sensors in the initial phase of the reaction. The observed rate decrease afterwards can be explained by diffusion of the gases from the liquid to the gas phase: As the liquid phase gas concentration increases, the concentration gradient between liquid and gas phases increases. This increased gradient leads to accelerated diffusion from the liquid to gas phase (following Fick's law), while the rate of gas production from water splitting likely remains relatively constant. Overall, this leads to a net decrease of the observed rate in the liquid phase. The liquid/gas phase mass transport can be controlled through the geometry of the reactor, specifically the size of the liquid/gas contact area as well as, to a certain extent, the liquid and gas phase volumes. This could be used to design experimental systems that are either optimized for liquid to gas phase diffusion that is slow (small contact area, little convection/mixing, high liquid phase volume, and small gas phase volume) or fast (large contact area, high convection/mixing, low liquid phase volume, large gas phase volume).

Secondly, the observed ratio of H_2_/O_2_ varies over the course of the experiment and only converges slowly to the expected 2:1 ratio in the gas phase. In Supporting Information Section 8.2, we provide a simple phenomenological model to rationalize this observation based on the different solubilities of H_2_ and O_2_ in water.

### 2.4 Kinetic Investigation of Overall Photocatalytic Water Splitting Using Rh_2*−y*_Cr_*y*_O_3_/Al:SrTiO_3_

With the data processing workflow established, we performed a series of experiments using the Rh_2*−y*_Cr_*y*_O_3_/Al:SrTiO_3_ photocatalyst. Initially, we established the reproducibility of the experimental and data processing protocol by testing the reference conditions (*T* = 20°C, 50 mW·cm^−2^ irradiance, 0.1 wt% Rh/Cr loading) seven times with liquid phase measurements. This gave maximum rates of H_2_ and O_2_ formation of 0.07 ± 0.01 and 0.05 ± 0.009 μmol·L^−1^·s^−1^ (error bars are standard deviations) in the liquid phase, respectively, showing good reproducibility of the measurements.

**FIGURE 3:**
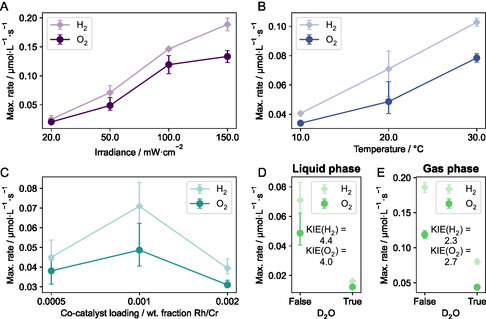
Impact of different experimental parameters on the maximum rates of H_2_/O_2_ formation (in the liquid phase, unless indicated otherwise). (A) Variation of irradiance. (B) Variation of reaction temperature. (C) Variation of cocatalyst loading. (D,E) Performing the reaction in either H_2_O or D_2_O to measure the kinetic isotope effect (KIE) using both liquid and gas phase measurements. The KIE is calculated based on the ratios of the maximum rates of both H_2_ and O_2_ formation. For all panels: shown error bars indicate the maximum and minimum rates obtained during experiments. The underlying experimental data is shown fully in Supporting Information Sections 8.3 and 8.5.

Next, we varied three experimental parameters to study their influence on the maximum rates in the liquid phase: irradiance, temperature, and cocatalyst loading (see Figure 3A–C).

Varying the irradiance from 20 to 150 mW·cm^−2^ (Figure 3A), we observed a roughly linear increase of the maximum rate. This first order dependence on irradiance is consistent with previous findings based on gas phase measurements [23, 26].

Increasing the temperature in the range of 10°C–30°C also results in an increase of the maximum rates (see Figure 3B), which indicates that there are rate‐limiting thermal reaction steps involved in the photocatalytic reaction sequence. Temperature dependence of the rate of photochemical reactions results from temperature‐dependent rate constants (analogous to thermal reactions), as described by Tolman [27], and the results are consistent with previous experimental findings for SrTiO_3_ photocatalysts [24, 28]. We performed an Arrhenius analysis (see Supporting Information Section 8.4) of the temperature‐dependent data and found thermal activation barriers of 33.1 ± 3.2 and 29.9 ± 3.0 kJ·mol^−1^ based on the H_2_ and O_2_ data, respectively.

Varying the loading of the Rh_2*−y*_Cr_*y*_O_3_ cocatalyst between 0.05 and 0.2 wt% (0.0005–0.002 wt. fraction, see Figure 3C) shows that the optimal loading is at 0.1 wt%, being also consistent with previous studies [26].

Finally, we performed the reaction also in D_2_O to measure the H/D kinetic isotope effect (KIE) (see Figure 3D,E). First, we performed the reaction with liquid phase measurements, finding KIEs of 4.4 and 4.0 based on the H_2_ and O_2_ data, respectively. The good agreement between the results based on H_2_ (D_2_) and O_2_ detection show that the different solubilities, diffusion coefficients, and possibly different response of the electrochemical sensor to D_2_ compared to H_2_ do not significantly impact the measurement (as there would otherwise be a larger difference to the KIE based on the O_2_ data, which is not affected by the switch from H_2_O to D_2_O). This rather large KIE would indicate that protons are involved in the rate‐determining step (H/D dependence of the rate constant for the rate determining step, analogous to temperature dependence of rate constant shown above). This, however, is in contrast to a previous finding in the literature, where Lercher and coworkers reported a KIE of 1.1 based on GC measurements [26]. Due to this deviation, we also performed analogous experiments with D_2_O and gas phase measurements (see Figure 3E). In this case, we found KIEs of 2.3 and 2.7 based on the H_2_ and O_2_ data, respectively. It is important to note that the maximum rates in the liquid and gas phases are observed on significantly different time scales: for liquid phase measurements, the maximum rate is observed after ca. 300 s, while for gas phase measurements, the maximum rate is observed after ca. 3,000–4,000 s. These different scales might point to a way to rationalize the different KIEs obtained in liquid and gas phase measurements as well as the result from the literature: Potentially, the rate‐determining step changes over the course of the reaction (e.g., due to different phases of catalyst activation/deactivation), with a rate‐determining step that involves protons in the initial reaction phase and a different rate‐determining step without proton involvement in the later reaction phase. Such a change over time could be related to the activation of the hydrogen evolution cocatalyst (Rh_2*−y*_Cr_*y*_O_3_), which was recently shown to undergo structural transformations on similar time scales during the initial reaction phase [29]. This rationalization, however, is only a hypothesis for now and requires further investigations. One possible experimental approach could be to systematically investigate the KIE at different reaction times to see if there is indeed a systematic trend. This could be combined with microkinetic modeling to understand which reaction steps contribute to the KIE at different times. Such investigations, however, are beyond the scope of the current work.

## 3 Conclusion

In summary, we report the design of a novel, open‐source photoreactor setup, which allows for reproducible overall water splitting experiments with in situ detection of H_2_ and O_2_ in both the liquid and gas phases. This layout allows for performing experiments under conditions that can be adapted to be analogous to scalable photoreactor systems, where the product gases accumulate in the gas phase. The presented measurement principle therefore gives *operando* insights which can be translated to application‐scale systems. Furthermore, the high time resolution offers rich kinetic insights into the overall water splitting reaction, which cannot be obtained using slower or gas phase methods such as GC.

We established a robust workflow for processing the experimental data obtained using this setup, yielding the maximum rates of H_2_ and O_2_ formation for both liquid and gas phase measurements. With the developed tools, we investigated the impacts of irradiance, temperature, and co‐catalyst loading on the maximum rates of overall water splitting using Rh_2*−y*_Cr_*y*_O_3_/Al:SrTiO_3_ and could successfully reproduce previous observations from the literature. Interestingly, measuring the H/D KIE in the liquid phase (4.0–4.4) and gas phase (2.3–2.7) gave different results, both of which are higher than those previously reported based on GC measurements. Due to the different time scales of liquid and gas phase measurements, this could possibly indicate a change in the rate determining step over the course of the reaction, but this hypothesis requires further investigation. Overall, the results highlight the versatility of the described approach for investigating overall photocatalytic water splitting, which will help to advance progress in the field.

## Author Contributions

**Nadzeya Brezhneva:** investigation, data curation, writing – original draft, writing – review and editing. **Alexander Eith:** investigation, data curation, writing – original draft, writing – review and editing. **Ebrahim Abedini:** investigation and data curation, writing – review and editing. **Daniel Kowalczyk:** investigation and data curation, writing – review and editing. **Dirk Ziegenbalg:** writing – review and editing, supervision, resources, funding acquisition. **Jacob Schneidewind:** conceptualization, writing – original draft, writing – review and editing, project administration, supervision, resources, funding acquisition. All authors have read and agreed to the published version of the manuscript.

## Supporting Information

Additional supporting information can be found online in the Supporting Information section. **Supporting Fig. S1:** SEM images of Al:SrTiO_3_ loaded with Rh_2−*y*_Cr_*y*_O_3_ cocatalyst at different magnifications. **Supporting Fig. S2:** Spectral distribution of the main elements in Al:SrTiO_3_ loaded with Rh_2−*y*_Cr_*y*_O_3_ photocatalyst (top), EDX overview of the sample (bottom). **Supporting Fig. S3:** XRD plot of the Al:SrTiO_3_ loaded with Rh_2−*y*_Cr_*y*_O_3_ cocatalyst. **Supporting Fig. S4:** BET N_2_ adsorption–desorption isotherm of Rh_2−*y*_Cr_*y*_O_3_/Al:SrTiO_3_ sample. **Supporting Fig. S5:** Irradiation setup with installed reactor and sensors. Left: complete irradiation setup, right: close‐up of the irradiation chamber. **Supporting Fig. S6:** Reactor used for irradiation experiments. Left: side view with shorter NS14 outlet in front, right: rotated view. **Supporting Fig. S7:** Calibration curve for Unisense sensor for H_2_ measurements in liquid phase. **Supporting Fig. S8:** Calibration curve for Unisense sensor for H_2_ measurements in gas phase. **Supporting Fig. S9:** Comparison of the results obtained from the measurements with Unisense H_2_ sensor and gas chromatography. **Supporting Fig. S10:** Important steps during photocatalytic tests: calibration of H_2_ sensor under degassed conditions (left), degassing of photocatalyst suspension (center), after start of irradiation, top view (right). **Supporting Fig. S11:** Emission spectrum of 365 nm LED. **Supporting Fig. S12:** Experimental data and kinetic modeling of H_2_/O_2_ evolution in the liquid (A) and gas phase (B). For both measurements, the evolution of the gases over time is shown (black and gray dots) as well as the ratio between the two gases (right axis, green dots). The concentration of the liquid phase gases is given in μmol·L^−1^ and the gas phase concentration is given in an analogous unit of μmol·L^−1^, which indicates the amount of gases formed per liter of irradiated liquid phase volume (to have consistent units for the kinetic modeling). To both the liquid and the gas phase data, one kinetic model (C) with two sets of rate constants fitted (black, gray and green lines). The optimized values for the rate constants are *k*_1_ = 4.1 × 10^−9^ s^−1^, *k*_2_ = 2.3 × 10^−3^ s^−1^, *k*_3_ = 2.2 × 10^−3^ s^−1^, and *k*_4_ = 5.8 × 10^−3^ s^−1^. **Supporting Fig. S13:** Experimental data for H_2_/O_2_ simultaneous measurements (reference conditions). **Supporting Fig. S14:** Experimental data for H_2_/O_2_ simultaneous measurements (screening of irradiance: 20, 100, and 150 mW·cm^−2^). **Supporting Fig. S15:** Experimental data for H_2_/O_2_ simultaneous measurements (screening of temperature: 10°C and 30°C). **Supporting Fig. S16:** Experimental data for H_2_/O_2_ simultaneous measurements (screening of cocatalyst loading: 0.0005 and 0.002 wt. fraction Rh/Cr). **Supporting Fig. S17:** Experimental data for H_2_/O_2_ simultaneous measurements (kinetic isotope effect investigation). **Supporting Fig. S18:** Experimental data for H_2_/O_2_ simultaneous measurements in gas phase (left: H_2_O as dispersion medium, right: D_2_O as dispersion medium for kinetic isotope effect investigation). **Supporting Fig. S19:** Arrhenius analysis of temperature‐dependent liquid phase experimental data to determine the thermal activation energy. **Supporting Table S1:** Overview of main reagents used for photocatalyst preparation and photocatalytic tests. **Supporting Table S2:** Overview of main equipment used for photocatalyst preparation and photocatalytic tests. **Supporting Table S3:** List of equipment used in photocatalytic tests for O_2_ and H_2_ measurements in liquid and gas phases. **Supporting Table S4:** Specifications of the hydrogen and oxygen sensors according to the respective manufacturer. **Supporting Table S5:** Phase shift difference for O_2_ sensor. **Supporting Table S6:** Comparison of hydrogen concentrations determined by Unisense H_2_ sensor and gas chromatography. **Supporting Table S7:** Overview of main screening parameters. **Supporting Table S8:** Classification of performed photocatalytic tests. **Supporting Table S9:** Maximum rates of H_2_ and O_2_ formation in performed photocatalytic tests.

## Funding

Financial support by Fonds der Chemischen Industrie (LiebigScholarship for J.S. and N.B.), Federal Ministry of Research, Technology and Space (BMFTR independent research group “SINATRA: SolSTEP”, grant number 03SF0729), and Deutsche Forschungsgemeinschaft (DFG) via the CRC TRR 234 CATALIGHT (project no. 36454990, projects A7 and C6) is gratefully acknowledged. Funding for the SEM instrument by the European Regional Development Fund (EFRE‐Programm 2021–2027 Thüringen; project no. 2023FGI0008, SEM@CEEC) is acknowledged.

## Conflicts of Interest

The authors declare no conflicts of interest.

## Code Availability Statement

Code developed for this study is available at: https://github.com/jschneidewind/simultaneous_detection.

## Supporting information

Supplementary Material

## Data Availability

Original data supporting the results of this study is available at https://github.com/water‐splitting‐group/o2_h2_reactor. This work has been previously posted as a preprint: https://doi.org/10.26434/chemrxiv‐2025‐q3fdh [30].
