# Supplementary material for: Insights Into Overall Photocatalytic Water Splitting Through Simultaneous In Situ H2 and O2 Measurements
Source: ChemSusChem. 2026 Mar 8;19(5):e202502721. doi: 10.1002/cssc.202502721 (PMC12967734; doi:10.1002/cssc.202502721)
Supplement: Supplementary file 1 — Supplementary Material [file CSSC-19-e202502721-s001.pdf]

# Supporting Information for

## Insights into overall photocatalytic water splitting through simultaneous *in situ* $H_2$ and $O_2$ measurements

Nadzeya Brezhneva, Alexander Eith, Ebrahim Abedini, Daniel Kowalczyk, Dirk Ziegenbalg, Jacob Schneidewind
Corresponding author(s), e-mail: jacob.schneidewind@uni-jena.de

**Other supplementary materials for this manuscript:**
Original data, analysis code and kinetic model code are publicly available at:
Github: https://github.com/water-splitting-group/o2_h2_reactor and https://github.com/jschneidewind/simultaneous_detection

# 1. Table of contents

**1. Table of contents** .......... **2**
1.1. List of figures .......... 3
1.2. List of tables .......... 5
**2. Experimental information** .......... **6**
2.1. Reagents and equipment .......... 6
**3. Photocatalyst preparation and characterization** .......... **8**
3.1. Calcination of SrCO3 .......... 8
3.2. Preparation of SrTiO3 .......... 9
3.3. Al doping of SrTiO3 .......... 9
3.4. RhCl3×3H2O and Cr(NO3)3×9H2O stock solutions preparation .......... 10
3.5. Loading of Al:SrTiO3 with Rh2-yCryO3 co-catalyst .......... 10
3.6. Photocatalyst characterization .......... 11
**4. Photocatalytic experiment hardware** .......... **15**
4.1. Irradiation setup and chamber .......... 15
4.2. Irradiation reactor .......... 16
4.3. Irradiance determination chamber .......... 17
4.4. Experimental setup for measurements in liquid and gas phase .......... 18
4.4.1. Specifications for H2 and O2 sensors, calibration data .......... 19
4.4.2. Potential error sources for H2 and O2 measurements .......... 21
4.4.3. Validation of H2 gas phase measurements by gas chromatography .......... 22
**5. Photocatalytic experiments with O2 and H2 simultaneous detection** .......... **24**
5.1. Photocatalyst suspension preparation .......... 24
5.2. Main steps in photocatalytic tests .......... 24
**6. Overview of performed photocatalytic tests** .......... **25**
**7. Analytical data** .......... **27**
7.1. Spectrum of light source .......... 27
**8. Data processing and analysis** .......... **27**
8.1. Detailed description of data processing workflow .......... 27
8.2. Liquid/gas phase mass transport during photocatalytic water splitting .......... 28
8.3. Processed experimental data .......... 30
8.4. Arrhenius analysis of temperature dependent data .......... 33
8.5. Overview of kinetic results .......... 34
**9. References** .......... **36**

## 1.1. List of figures


- Figure 1: SEM images of Al:$SrTiO_3$ loaded with $Rh_{2-y}Cr_yO_3$ co-catalyst at different magnifications
- Figure 2: Spectral distribution of the main elements in Al:$SrTiO_3$ loaded with $Rh_{2-y}CryO_3$ photocatalyst (top), EDX overview of the sample (bottom).
- Figure 3: XRD plot of the Al:$SrTiO_3$ loaded with $Rh_{2-y}Cr_yO_3$ co-catalyst.
- Figure 4: BET $N_2$ adsorption - desorption isotherm of $Rh_{2-y}Cr_yO_3$/Al:$SrTiO_3$ sample
- Figure 5: Irradiation setup with installed reactor and sensors. Left: complete irradiation setup, right: close-up of the irradiation chamber
- Figure 6: Reactor used for irradiation experiments.Left: Side view with shorter NS14 outlet in front, right: rotated view
- Figure 7: Calibration curve for Unisense sensor for $H_2$ measurements in liquid phase
- Figure 8: Calibration curve for Unisense sensor for $H_2$ measurements in gas phase
- Figure 9: Comparison of the results obtained from the measurements with Unisense $H_2$ sensor and gas chromatography.
- Figure 10: Important steps during photocatalytic tests: calibration of $H_2$ sensor under degassed conditions (left), degassing of the photocatalyst suspension (center), after start of irradiation, top view (right)
- Figure 11: Emission spectrum of 365 nm LED.
- Figure 12: Experimental data and kinetic modelling of $H_2/O_2$ evolution in the liquid (A) and gas phase (B). For both measurements the evolution of the gases over time is shown (black and grey dots) as well as the ratio between the two gases (right axis, green dots). The concentration of the liquid phase gases is given in $\mu mol.L^{-1}$ and the gas phase concentration is given in an analogous unit of $\mu mol.L^{-1}$, which indicates the amount of gases formed per liter of irradiated liquid phase volume (to have consistent units for the kinetic modelling). To both the liquid and the gas phase data one kinetic model (C) with one set of rate constants is fitted (black, grey and green lines). The optimized values for the rate constants are: $k_1$ = $4.1.10^{-9}$ $s^{-1}$, $k_2$ = $2.3.10^{-3}$ $s^{-1}$, $k_3$ = $2.2.10^{-3}$ $s^{-1}$, $k_4$ = $5.8.10^{-3}$.
- Figure 13: Experimental data for $H_2/O_2$ simultaneous measurements (reference conditions)
- Figure 14: Experimental data for $H_2/O_2$ simultaneous measurements (screening of irradiance: 20 $mW.cm^{-2}$, 100 $mW.cm^{-2}$, 150 $mW.cm^{-2}$)
- Figure 15: Experimental data for H2/O2 simultaneous measurements (screening of temperature: 10 °C, 30 °C)
- Figure 16: Experimental data for $H_2/O_2$ simultaneous measurements (screening of co-catalyst loading: 0.0005 wt. fraction Rh/Cr, 0.002 wt. fraction Rh/Cr)
- Figure 17: Experimental data for $H_2/O_2$ simultaneous measurements (kinetic isotope effect investigation)
- Figure 18: Experimental data for $H_2/O_2$ simultaneous measurements in gas phase (left: $H_2O$ as a dispersion medium, right: $D_2O$ as a dispersion medium for kinetic isotope effect investigation)

- Figure 19: Arrhenius analysis of temperature dependent liquid phase experimental data to determine the thermal activation energy

## 1.2. List of tables


- Table 1: Overview of main reagents used for photocatalyst preparation and photocatalytic tests
- Table 2: Overview of main equipment used for photocatalyst preparation and photocatalytic tests
- Table 3: List of the equipment used in the photocatalytic tests for $O_2$ and $H_2$ measurements in liquid and gas phase
- Table 4: Specifications of the used sensors for hydrogen and oxygen sensor according to the respective manufacturer.
- Table 5: Phase shift difference for $O_2$ sensor
- Table 6: Comparison of the hydrogen concentrations determined Unisense $H_2$ sensor and gas chromatography
- Table 7: Overview of main screening parameters
- Table 8: Classification of performed photocatalytic tests
- Table 9: Maximum rates of $H_2$ and $O_2$ formation in performed photocatalytic tests

# 2. Experimental information

## 2.1. Reagents and equipment

List of the chemicals used in work is presented in table 1 below:

Table 1: Overview of main reagents used for photocatalyst preparation and photocatalytic tests

| Name of the reagent, purity | Supplier |
|---|---|
| $SrCO_3$, > 98.0% | TCI |
| $TiO_2$, Aeroxide® P25 | Thermo Scientific |
| $SrCl_2 \times 6H_2O$, 99% (ACS) | Strem Chemicals |
| $AgNO_3$ | VEB Feinchemie Sebnitz |
| $RhCl_3 \times 3H_2O$ | BLD Pharmatech GmbH |
| $Cr(NO_3)_3 \times 9H_2O$ | Acros Organics |
| Ethanol, 96% | / |
| $D_2O$, 99.90 % | Eurisotop |

In all experiments milli-Q water (18.2 MΩ.cm) was used.

A list of the used equipment is provided in table 2 below.

Table 2: Overview of main equipment used for photocatalyst preparation and photocatalytic tests

| Name of the step | Model of the device | Supplier |
|---|---|---|
| Photocatalyst preparation | Muffle furnace Nabertherm LT 15/11/P330 | Nabertherm GmbH |
| | Muffle furnace Nabertherm L3/11/P320 | Nabertherm GmbH |
| | Drying oven Binder FD 56 E3.1 | Binder GmbH |
| | Ultrasound bath ELMA Fischerbrand Select 30 | Fischer Scientific |
| | Magnetic stirrer Heidolph Instruments Hei-PLATE Mix'n'Heat Core+Ø135 | LT Laborhandel GmbH |
| | Vortex mixer VV3 | VWR |
| Irradiation | Double walled beaker | Glassblower |
| | Thermostat LAUDA LOOP L 100 | LAUDA |

| | | |
|---|---|---|
| | Ultra-high power LED LCS-6500-65-22, 365 nm | Mightex Systems |
| | Power meter Newport 843-R-USB | Newport Spectra-Physics GmbH |
| | Thermopile sensor Newport 919P-020-12 | Newport Spectra-Physics GmbH |
| Gas and liquid phase $O_2$ detection | Trace range robust probe TROXROB-CV | PyroSience GmbH |
| | Firesting Fiber-Optic Oxygen Meter 2 Channel | PyroSience GmbH |
| | PT100 temperature sensor | Therma Thermofühler GmbH |
| Gas and liquid phase $H_2$ detection | $H_2$ UniAmp Single Channel system (amplifier) | Unisense |
| | Normal range hydrogen sensor $H_2$ UniAmp Sensor - Normal range - 2.1 x 80 mm needle | Unisense |
| | PT1000 temperatures sensor | Therma Thermofühler GmbH |
| Photocatalyst characterization | TESCAN Mira scanning electron microscope | TESCAN |
| | XRD-BRUKER-D2 PHASER diffractometer | Bruker |
| | Volumetric gas sorption analyzer Quantachrome Quadrasorb, version 3.0 | Quantachrome Instruments |

Scanning electron microscopy (SEM) of the Al:$SrTiO_3$ loaded with $Rh_{2-y}Cr_yO_3$ (0.1 wt% Rh,Cr) photocatalyst was performed using a TESCAN MIRA microscope equipped with a secondary electron detector and operated at an accelerating voltage of 5 kV. The sample powder was mounted on conductive carbon adhesive pads attached to aluminum pin stubs. Energy-dispersive X-ray (EDX) spectroscopy was carried out using an Essence™ EDS system equipped with an integrated detector featuring a 30 mm$^2$ active area and a $Si_3N_4$ window, providing an energy resolution of 129 eV at the Mn Kα line.

XRD measurements of the photocatalyst were performed using XRD-BRUKER-D2 PHASER diffractometer.

The measurements were performed with the following parameters:

1. Tube: Cu tube with 1.54184 [Å],
2. Detector: SSD160_2 (1D mode),
3. 2θ start from 5°, stop at 100°, Increment: 0.02°,
4. 1.00 sec per step, 4701 steps, Total time: 4801 sec.
5. Scan type: Coupled 2θ/θ,
6. Scan mode: Continuous PSD fast.
7. Sample holder: Specimen stainless steel silicon ground

The Brunauer-Emmett-Teller (BET) surface area was determined by $N_2$ physisorption using a volumetric gas sorption analyzer (Quantachrome Quadrasorb, Quantachrome Instruments, version 3.0) at −196 °C. Prior to the measurements, the sample was outgassed under vacuum at 150 °C for 15 h.

# 3. Photocatalyst preparation and characterization

Preparation of Al:$SrTiO_3$ loaded with $Rh_{2-y}Cr_yO_3$ co-catalyst was inspired by the group of Prof. F. Osterloh [1]. Initially, $SrTiO_3$ was prepared via solid-state synthesis from $SrCO_3$ and $TiO_2$ (1000 °C, 10 h), followed by Al-doping procedure in alumina crucibles in the presence of $SrCl_2$ (1000 °C, 10 h) used as a flux medium . Afterwards, the obtained product was washed with milli-Q water (to remove $SrCl_2$ till no $Cl^-$ could be detected with 0.1 M $AgNO_3$ solution) and dried at 100 °C overnight. The co-catalyst loading was performed *via* wet impregnation method using $RhCl_3$×$3H_2O$ and $Cr(NO_3)_3$×$9H_2O$ solutions to achieve 0.1 wt% Rh and Cr in the final product. The detailed description of each step of material synthesis is provided below.

## 3.1. Calcination of $SrCO_3$

Before preparation of $SrTiO_3$, calcination of $SrCO_3$ was required.
$SrCO_3$ (9.31 g) was weighed on a weighing boat and transferred to an agate mortar. The sample was mortared for 2 min and transferred into a porcelain crucible. The crucible covered with lid was transferred into the Nabertherm Muffle furnace L3/11/P320 (Nabertherm GmbH) and the heating program was started (300 °C, 1 h, heating rate 10 °/min). After calcination was finished, the sample was removed from the furnace and weighed. The sample was a white powder, m = 9.24 g, yield 99.2%.

## 3.2. Preparation of $SrTiO_3$

Freshly calcined $SrCO_3$ (6.21 g, 42 mmol, 1 equiv.) was weighed in a weighing boat, $TiO_2$ (3.37 g, 42 mmol, 1 equiv.) was weighed in another weighing boat. The weighed samples were transferred to an agate mortar and mixed for approx. 10 min. During mixing, 250 µL of EtOH was added to the mixture and the mortaring of solid was continued. The procedure of EtOH addition was repeated 3 more times.

After mortaring, the mixture of solids was transferred to a 150 mL alumina crucible. The mixture was pressed with an agate pestle (without applying force, just to make the solid mixture a bit more compact inside the crucible).

The crucible covered with lid was transferred to a Nabertherm LT15/11/P330 (Nabertherm GmbH) muffle furnace and the heating program was started (1000 °C, 1 h, heating rate 10 °/min). After calcination was finished, the solid from the crucible was transferred to a weighing bowl and the lumps were broken into smaller pieces with plastic spatula to make the solid more homogeneous. The solid after calcination was represented with white solid, m = 7.64 g, yield = 99.0 %.

## 3.3. Al doping of $SrTiO_3$

$SrCl_2 \times 6H_2O$ (79.95 g, 299.86 mmol, 10 equiv.) was weighed in a bowl. $SrTiO_3$ (5.55 g, 30.246 mmol, 1 equiv.) was weighed in a weighing bowl. The materials were transferred to an agate mortar and the mixture was mortared for approx. 15 min. The mortared solid was distributed between three 150 mL alumina crucibles. The mixture inside the crucible was pressed with an agate pestle (without applying force, just to make the solid mixture a bit more compact inside the crucible).

The crucibles covered with lid was transferred to a Nabertherm LT15/11/P330 (Nabertherm GmbH) muffle furnace and the heating program was started (1000 °C, 10 h, heating rate 10 °/min). After cooling down, the crucibles were removed.

25 mL of water was added to each alumina crucible via a graduated cylinder. The suspension inside the crucible was scratched with plastic spatula, afterwards the suspension was left for 1 h for better dissolution of $SrCl_2$ and further transfer of the calcined solid from the crucible.

After 1 h, the suspension was sonicated in an ultrasonic bath for 15 s. Next, the suspension was transferred to a 600 mL glass beaker. To remove the total amount of calcined solid, the crucibles were filled with 25 mL water one more time and left for approx. 10-15 min. Afterwards, the samples were sonicated in an ultrasonic bath and transferred to the 600 mL glass beaker. This was repeated 4 more times to transfer the solid from the crucible quantitatively.

Next, the sample was filtered using a PVDF filter (0.22 µm pore diameter). Afterwards, the collected solid on the top of the filter was thoroughly washed with water (70 °C), carefully scratching the slurry to provide thorough washing of the solid. Periodically, $Cl^-$ tests with the use of 0.1 M $AgNO_3$ solution were performed (in total, 16 tests were performed till the

solution after mixing with 0.1 M $AgNO_3$ became clear and overall approx. 10 L of water for washing procedure was used).
The washed solid on the top of the PVDF filter was transferred to a Petri dish by creating a slurry (roughly, approx. 30 mL of water was added to a PVDF filter, the solid was carefully scratched from the surface to create a slurry and transfer it afterwards to a Petri dish using a glass pipette). The Petri dish was covered with Al foil with perforated holes in it. The dish was transferred to a drying oven (100 °C, 12 h). Afterwards, the dried sample was collected from the Petri dish and weighed. The final product (Al:$SrTiO_3$) was represented with creamy solid, m = 5222.61 mg, yield = 94.1 %.

## 3.4. $RhCl_3$×3$H_2O$ and $Cr(NO_3)_3$×9$H_2O$ stock solutions preparation

$RhCl_3$×3$H_2O$ (13 mg, 0.049 mmol) was weighed on a weighing paper and transferred to a 10 mL vial. $Cr(NO_3)_3$×9$H_2O$ (57.64 mg, 0.144 mmol) was weighed in a 10 mL vial. Water (2000 µL) was added to each vial to dissolve the solids. The solutions were transferred to a 15 mL Falcon tube. 1000 µL of water was added to each vial to transfer the solution from the vial quantitatively to the Falcon tube. The volume of each solution was adjusted to 5 mL by gradual addition of water till the constant volume.

## 3.5. Loading of Al:$SrTiO_3$ with $Rh_{2-y}Cr_yO_3$ co-catalyst

Al:$SrTiO_3$ (500.12 mg, 2.725 mmol) was weighed on a weighing paper and transferred to a 100 mL borosilicate 3.3 beaker. Water (12.5 mL) was transferred to the beaker and stirring (300 rpm) was started. $RhCl_3$×3$H_2O$ solution (492 µL, 9.874 mM) was added fast to the suspension under stirring, afterwards $Cr(NO_3)_3$×9$H_2O$ solution (334 µL, 28.809 mM) was added fast to the suspension under stirring. The mixture was stirred for ca. 3 min for better distribution of the components.
The beaker was transferred to a water bath (70 °C). After evaporation of the total amount of water, the stirring and heating was stopped.
The crystallizing dish (borosilicate glass, d = 8 cm) was placed on a beaker as a lid. The beaker covered with lid was transferred to the Nabertherm Muffle furnace L3/11/P320 (Nabertherm GmbH) and the heating program was started (350 °C, 1 h, heating rate 10 °/min). After calcination was finished, the solid from the beaker was collected from the walls and the bottom of the beaker and weighed. The final product was represented with light grey-pinkish solid, m = 495.98 mg, yield = 99.2 %.

For preparation of the photocatalyst with 0.05 wt% Rh and Cr content in the final product the following procedure was applied.
Al:$SrTiO_3$ (149.51 mg, 0.81 mmol) was weighed on a weighing paper and transferred to a 25 mL borosilicate beaker afterwards. Water (3876 µL) was transferred to the beaker, placed on the magnetic stirrer (Heidolph) and the stirring was started (400 rpm). $RhCl_3$×3$H_2O$ solution (74 µL, 9.874 mM) was added to the suspension under stirring, afterwards $Cr(NO_3)_3$×9$H_2O$

solution (50 µL, 28.809 mM) was added to the suspension under stirring. The mixture was stirred for ca. 3 min for better distribution of the components.

The beaker was transferred to a water bath equipped with an external temperature sensor from the magnetic stirrer, afterwards, the heating was started (70 °C, precise mode). After evaporation of the total amount of water (ca. 2 h), the stirring and heating were stopped. The dried solid was transferred to a quartz crucible using Smartspatula. The quartz crucible covered with lid was placed in Nabertherm muffle furnace L3/11/P320 (Nabertherm GmbH), and the heating program was started (350 °C, 1 h, heating rate 10 °/min). After calcination was finished, the solid from the crucible was weighed. The final product was represented with creamy-greyish solid, m = 142.14 mg, yield = 95.1 %.

For preparation of the photocatalyst with 0.2 wt% Rh and Cr content in the final product the following procedure was applied.

Al:$SrTiO_3$ (152.05 mg, 0.83 mmol) was weighed on a weighing paper and transferred to a 25 mL borosilicate beaker afterwards. Water (3505 µL) was transferred to the beaker, placed on the magnetic stirrer (Heidolph) and the stirring was started (400 rpm). $RhCl_3 \times 3H_2O$ solution (295 µL, 9.874 mM) was added to the suspension under stirring, afterwards $Cr(NO_3)_3 \times 9H_2O$ solution (200 µL, 28.809 mM) was added to the suspension under stirring. The mixture was stirred for ca. 3 min for better distribution of the components.

The beaker was transferred to a water bath (Benmari-crystallizing dish 8cm filled with water) equipped with an external temperature sensor from the magnetic stirrer. The heating was started (70 °C, precise mode). After evaporation of the total amount of water (ca. 2 h), the stirring and heating was stopped. The dried solid was transferred to a quartz crucible using Smartspatula. Quartz crucible covered with lid was placed in Nabertherm muffle furnace L3/11/P320 (Nabertherm GmbH). and the heating program was started (350 °C, 1 h, heating rate 10 °/min). After calcination was finished, the solid from the crucible was weighed. The final product was represented with creamy-greyish solid, m = 142.57 mg, yield = 93.8 %.

## 3.6. Photocatalyst characterization

The morphology of the obtained photocatalyst is presented in Figure 1.

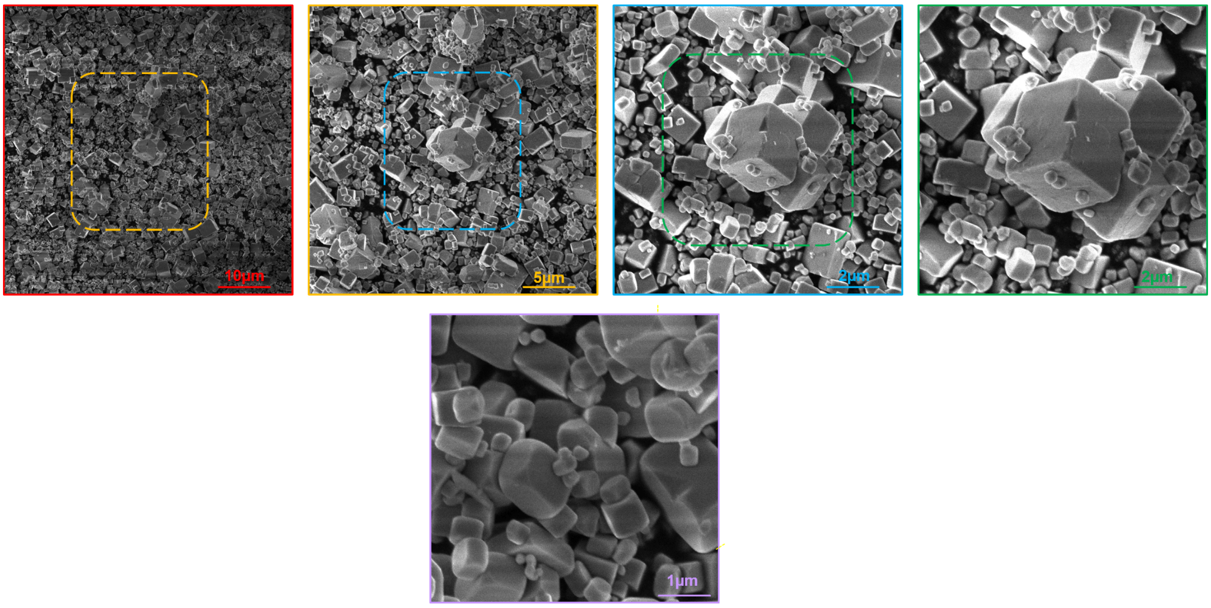


Figure 1: SEM images of $Al:SrTiO_3$ loaded with $Rh_{2-y}Cr_yO_3$ co-catalyst at different magnifications

The truncated, cubic-like crystals were formed after Al flux treatment. These crystals with specific facets appear in different sizes. These cube-shaped particles can be seen with edge sizes between 500 nm to 2 µm. The majority of the particles were within the 0.5 µm range, whereas the larger particles appeared partially.

Results of EDX analysis with the spectral distribution of the main elements (O, Sr, Ti, Al, Rh, Cr) of the sample and mapping are presented in Figure 2.

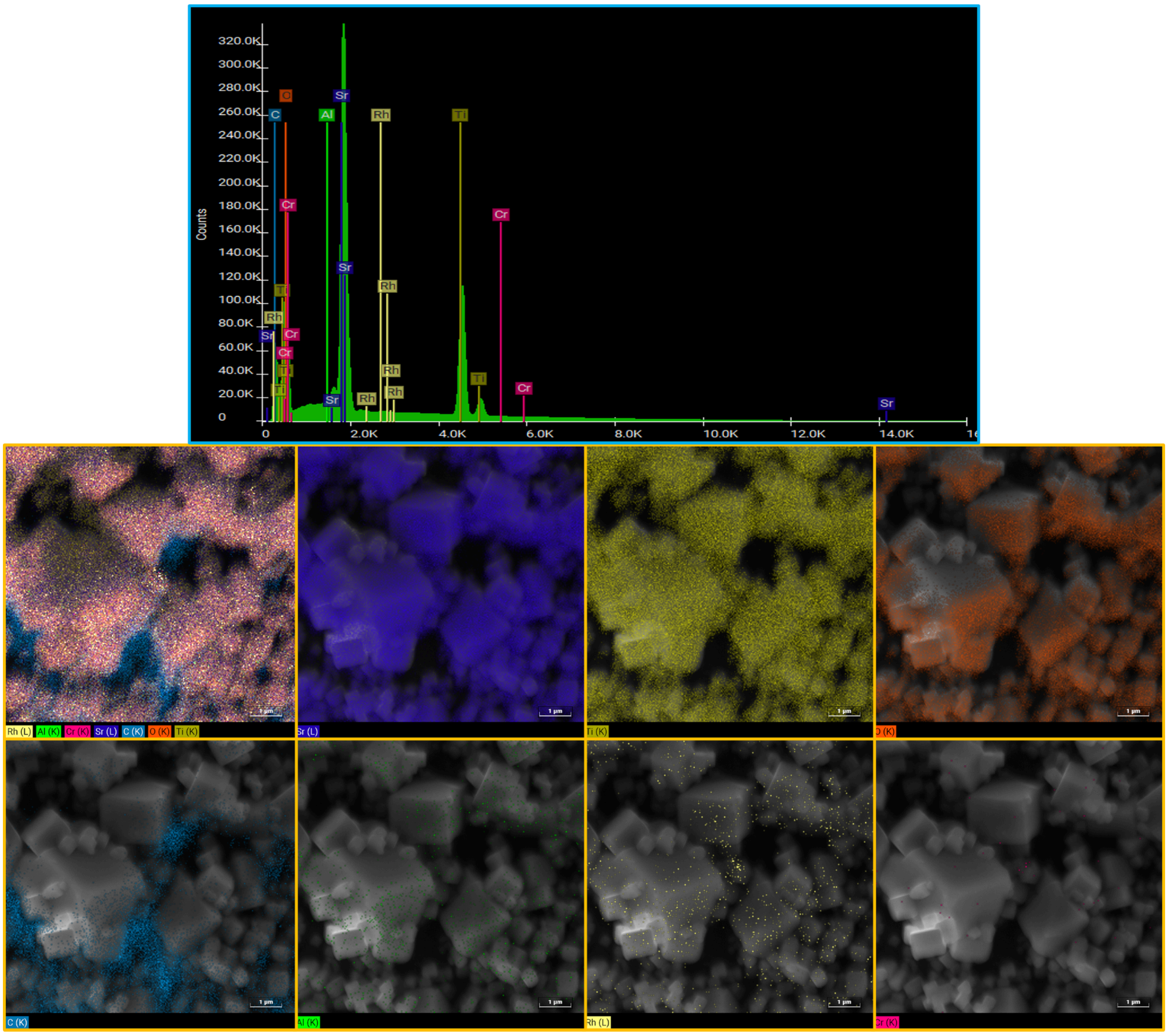


Figure 2: Spectral distribution of the main elements in Al:$SrTiO_3$ loaded with $Rh_{2-y}Cr_yO_3$ photocatalyst (top), EDX overview of the sample (bottom).

EDX analysis confirmed the formation of $SrTiO_3$ as a main phase, the presence of Al in the sample from the doping process and Rh and Cr from the wet impregnation step. The signal of C comes from the conductive carbon tape.

Diffractogramm of the photocatalyst is presented in Figure 3.

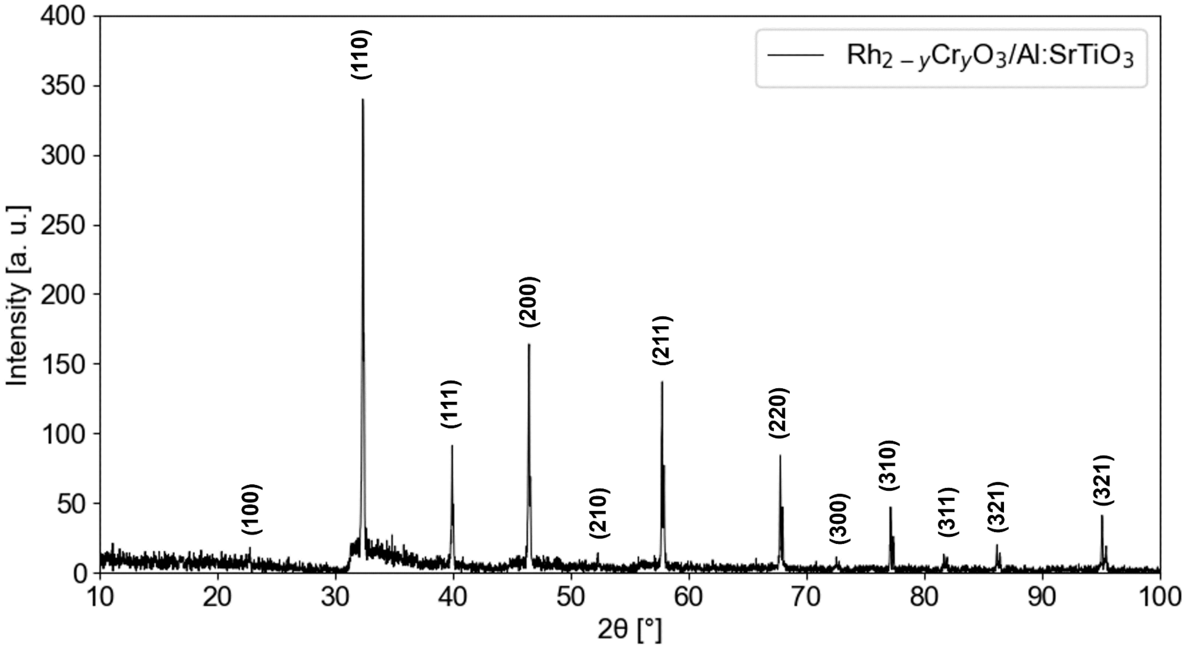


Figure 3: XRD plot of the Al:$SrTiO_3$ loaded with $Rh_{2-y}Cr_yO_3$ co-catalyst.

The structure is in good agreement with cubic perovskite structure of $SrTiO_3$ according to JCPDS Card No. 35-0734[2] .

Figure 4 illustrates the nitrogen adsorption–desorption isotherm of $Rh_{2-y}Cr_yO_3$/Al:$SrTiO_3$. Based on the SEM observations and XRD analysis, the sample exhibits a non-porous structure. Accordingly, the isotherm does not show a pronounced hysteresis loop and can be classified as a Type II isotherm. This behavior is in good agreement with the morphology and crystallinity of the synthesized photocatalyst.

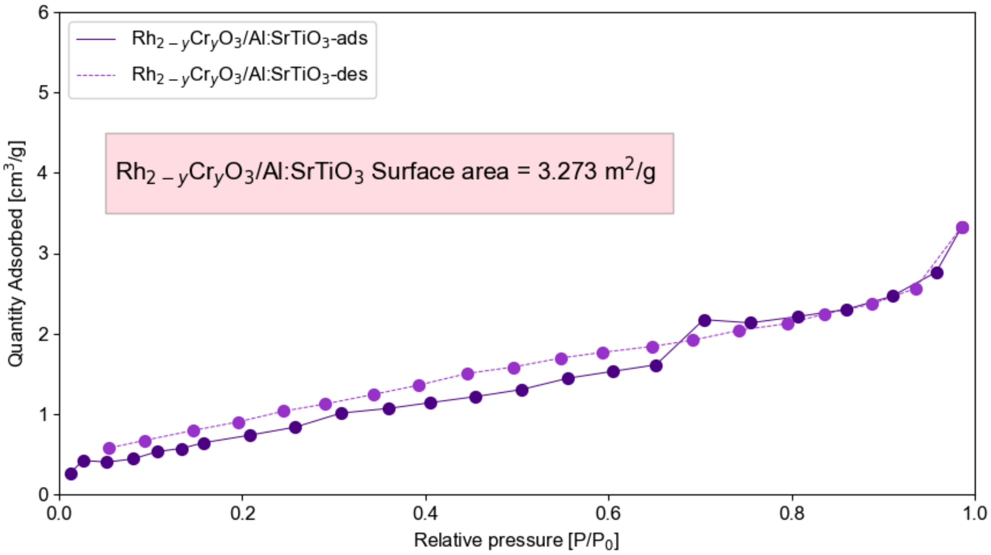


Figure 4: $N_2$ adsorption - desorption isotherm of $Rh_{2-y}Cr_yO_3$/Al:$SrTiO_3$.

The BET surface area of the sample was determined to be 3.273 $m^2.g^{-1}$, which is in good agreement with previously reported values.[3]

# 4. Photocatalytic experiment hardware

## 4.1. Irradiation setup and chamber

The setup consisted of a black, wooden box (L x W x H: 50 x 45 x 70 cm), mounted on black aluminium profiles (40×40 mm, light-duty, slot 8, I-type). A 15 x 15 cm opening (25 cm above the base) was cut into the back wall to allow routing of tubing and cables. Inside the enclosure, two lab jacks and a laboratory stand were installed. The 365 nm LED was placed on one lab jack and a magnetic stirring plate on the other. An irradiation chamber inspired by the design in [4] was positioned on the stirring plate (see Figure 5, left).

The chamber was fabricated from polylactic acid using fuse-deposition modelling, employing black filament (Primacreator, Primavalue PLA+ black, 1.75 mm filament size) to minimize light reflection and scattering. The internal dimensions were 130 x 130 x 130 mm. The chamber comprised two side walls with horizontal slots for the reactor holder, as well as an aperture where on one side a solid dummy aperture was installed and on the other side an irradiation aperture. The back wall contained a cutout for water-cooling tubing. In the bottom an aperture for the double-walled beaker was placed (see Figure 5, right).

The double-walled beaker had an inner diameter of approx. 45 mm and an outer diameter of approx. 60 mm, with inner and outer heights of approx. 50 and 60 mm. Opposing inlet (bottom) and outlet (top) were connected to a thermostat (Lauda Loop 100). Both the inner compartment and the interspace between the two walls were filled with Milli-Q water. The aperture for the double-walled beaker was designed in such a way that the center of the light beam was at the center of the double walled beaker.

The reactor was secured in the double-walled beaker using a holder placed in the uppermost slot of the chamber walls. The distance between the 365 nm LED and the center of the reactor was 80 mm.

All CAD drawings and STL files of the irradiation chamber, as well as the assembly description and a sketch of the double walled beaker are available in the referenced GitHub repository and as a stable release on Zenodo.

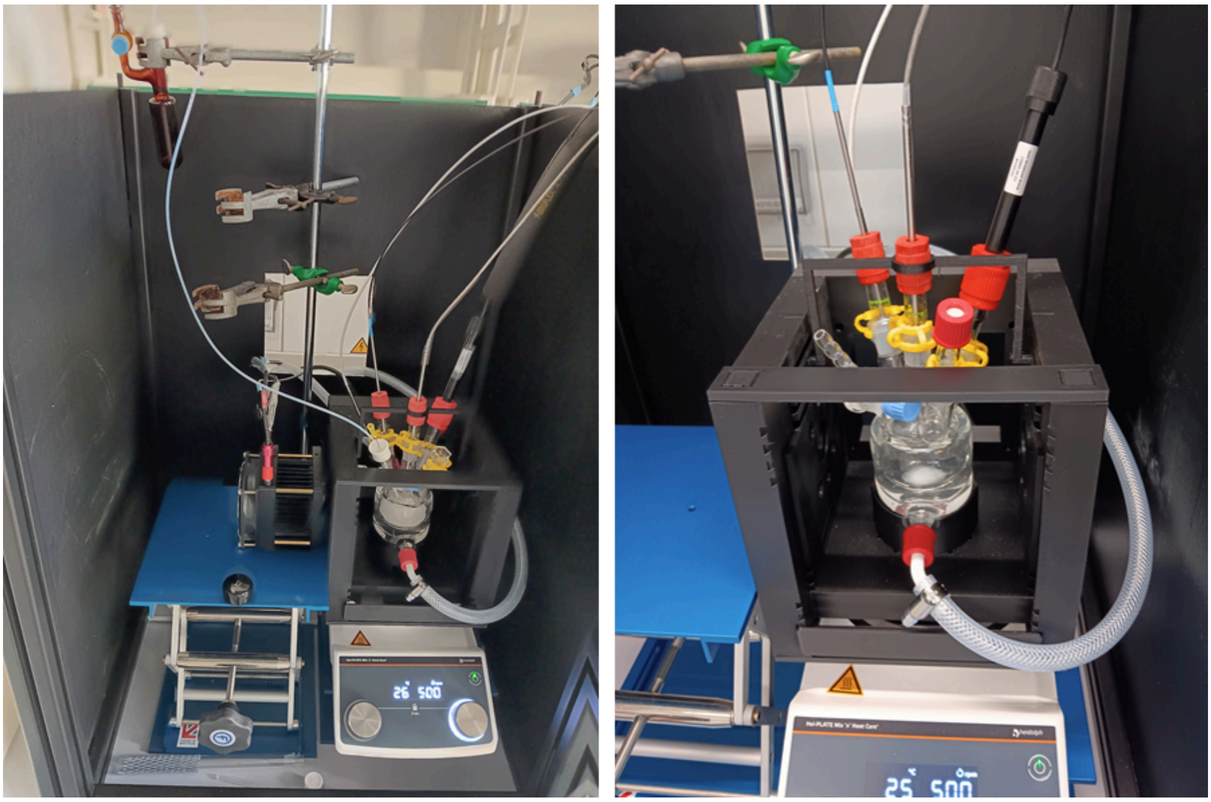

Figure 5: Irradiation setup with installed reactor and sensors. Left: complete irradiation setup, right: close-up of the irradiation chamber

## 4.2. Irradiation reactor

The reactor was custom built by a glassblower. The main body (see Figure 6) had a rounded bottom with an outer diameter of 40 mm and a height of approx. 40 mm up to the beginning of the necks. Five outlets were fused onto the top in a cross-shaped arrangement. One outlet contained a valve connection (NS14.5 with a 4 mm bore). The remaining four outlets were NS14 outer joints. For three of these, the total height from the lowest attachment point to the top was approx. 60 mm; the fourth — positioned opposite the valve — was shorter (< 50 mm, see Figure 5).

Each NS14 joint was equipped with a GL14 or GL18 transition adapter. The total adapter height was approx. 60 mm for GL14 and < 50 mm for GL18.

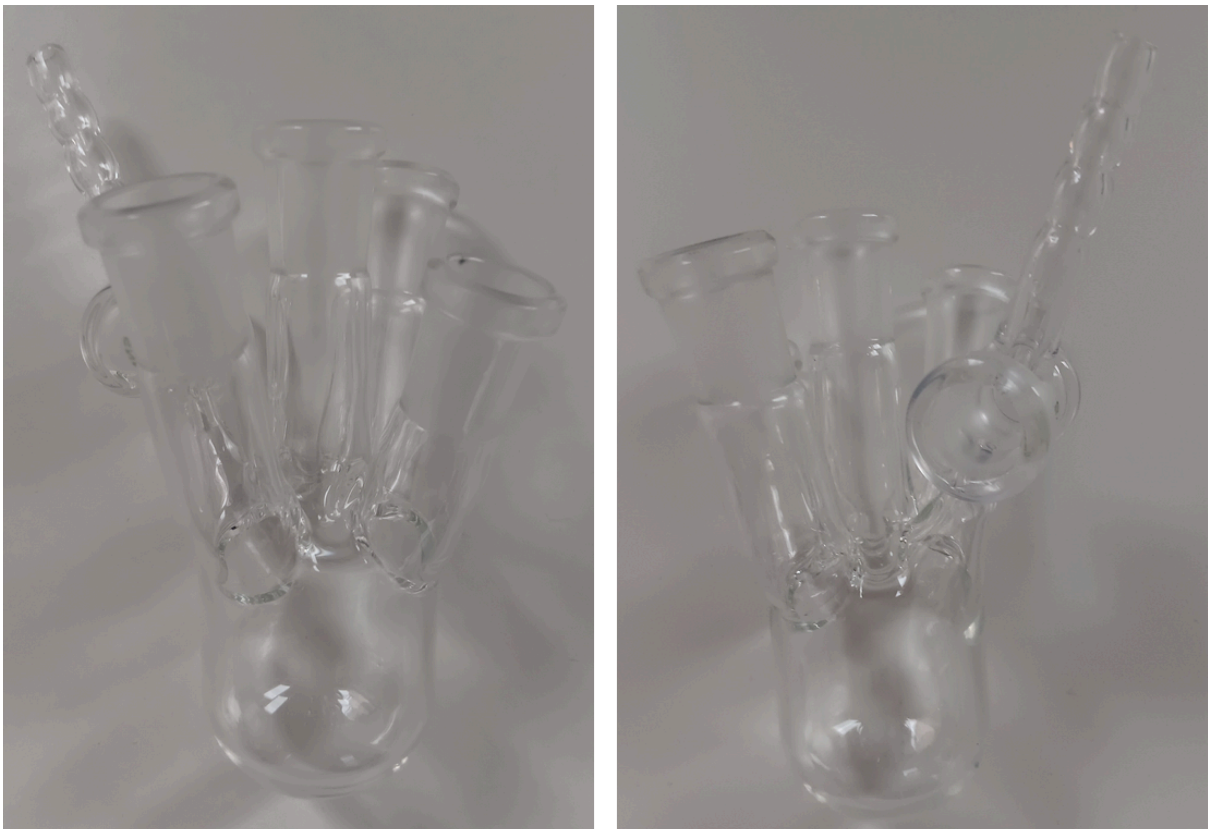

Figure 6: Reactor used for irradiation experiments.Left: Side view with shorter NS14 outlet in front, right: rotated view

## 4.3. Irradiance determination chamber

Irradiance was measured in a dedicated irradiation chamber similar to the one described in 4.1. but modified as follows:

- Solid back wall without cutout
- Solid front wall
- Removable lid on top

These modifications ensured minimal light leakage and allowed the use independently of the full setup.
For irradiance measurements, the power sensor was positioned in the center of the lightbeam using either a holder, inserted into the lowest wall slot or an aperture which was placed into the bottom.
All CAD drawings and STL files of the irradiance determination chamber, as well as the assembly description are available in the referenced GitHub repository and as a stable release on Zenodo.

## 4.4. Experimental setup for measurements in liquid and gas phase

Summary of the devices used in photocatalytic tests is provided in table 3 below.

Table 3: List of the equipment used in the photocatalytic tests for $O_2$ and $H_2$ measurements in liquid and gas phase

| Name of the step | Name of the device |
|---|---|
| Measurements of optical power output | Super high-power 365 nm LED collimator source with a 22 mm clear aperture (LCS-6500-65-22, Mightex Systems) (365 nm LED) |
| | Newport 843-R-USB power meter (Newport) |
| | Newport 919P-020-12 thermopile sensor (Newport) |
| $O_2$ measurements (liquid and gas phase) | FireStingO2 optical oxygen meter (FSO2-C2, PyroSience GmbH) |
| | Trace range robust probe (TROXROB-CV, PyroSience GmbH) in combination with 3 mm BOLA fitting and GL14/NS14 adapter |
| | PT100 temperature sensor (Therma Thermofühler GmbH) in combination with 4 mm BOLA fitting and GL14/NS14 adapter |
| $H_2$ measurement (liquid phase) | $H_2$ UniAmp Single Channel system (Unisense) |
| | Normal range hydrogen sensor ($H_2$ UniAmp Sensor - Normal range - 2.1 x 80 mm needle, Unisense) in combination with 10 mm BOLA fitting and GL18/NS14 adapter |
| | PT1000 temperature sensor (Therma Thermofühler GmbH) in combination with 4 mm BOLA fitting and GL14/NS14 adapter |
| $H_2$ measurements (gas phase) | $H_2$ UniAmp Single Channel system (Unisense) |

| | |
|---|---|
| | Normal range hydrogen sensor ($H_2$ UniAmp Sensor - Normal range - 2.1 x 80 mm needle, Unisense) in combination with 2 mm BOLA fitting and GL14/NS14 adapter |
| | PT1000 temperature sensor (Therma Thermofühler GmbH) in combination with 4 mm BOLA fitting and GL14/NS14 adapter |
| | Gas chromatograph Shimadzu Nexis GC-2030 |

Photochemical irradiations were performed using the 365 nm LED (For the emission spectrum, see chapter 7.1).
Before each experiment series, the irradiance was measured using the irradiance determination chamber (see section 4.3.). Measurements were repeated approx. weekly to account for minor fluctuations in the LED output. The hydrogen sensor was calibrated daily by bubbling hydrogen gas through the solution, as its sensitivity changes over time.
The sensors were installed using gas-tight BOLA laboratory joints. The PT1000 was placed centrally using a 4 mm BOLA joint, after which the reactor was secured in its holder. The PT100 and the FireSting robust probe were installed using 4 and 3 mm BOLA joints, respectively.

### 4.4.1. Specifications for $H_2$ and $O_2$ sensors, calibration data

The specifications of the used hydrogen and oxygen sensors according to the respective manufacturer (see chapter 4.4) are stated in table 4.

Table 4: Specifications of the used sensors for hydrogen and oxygen sensor according to the respective manufacturer.

| **Specification** | **Unisense hydrogen sensor** | | **Pyroscience oxygen sensor** | |
|---|---|---|---|---|
| | Gas phase | Liquid phase | Gas phase | Liquid phase |
| Lower detection limit | 0.04 % | 0.3 µM | 0.005 % | 0.1 µM |
| Upper detection limit | 250 % | 2000 µM | 10 % | 500 µM |
| Resolution | not stated | not stated | 0.002 % | 0.05 µM |
| Accuracy | not stated | not stated | 2 % | 2 % |
| Response time | <15 s | <15 s | <3 s | <20 s |

Prior to photocatalytic experiments, the linearity of the Unisense $H_2$ sensor response was verified. For this purpose, the reactor containing water was initially purged with argon for 20 min and the 0 ppm signal was recorded. Subsequently, 0.25 mL of hydrogen-saturated water was added, corresponding to a hydrogen concentration of 9901 ppm (relative to hydrogen-saturated water). After signal stabilization, the sensor response was recorded. Next, 1.00 mL of hydrogen-saturated water was added, yielding a concentration of 47619 ppm, and the stabilized signal was again recorded. Finally, the reactor solution was bubbled with hydrogen-saturated water to obtain the $10^6$ ppm data point, which was likewise recorded after signal stabilization (see Figure 7).

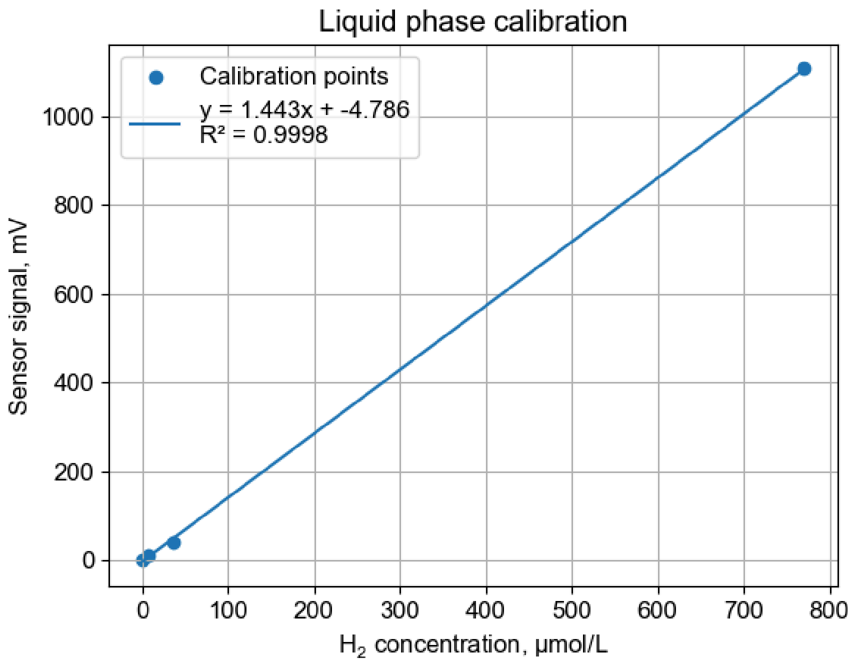


Figure 7: Calibration curve for Unisense sensor for $H_2$ measurements in liquid phase

To evaluate the sensor response in the gas phase, water-saturated hydrogen was added stepwise to the reactor in volumes of 0.06, 0.56, 1.00, 2.0, and 3.0 mL, resulting in hydrogen concentrations of 0, 1056, 10912, 28511, 63710, and 118508 ppm, respectively. After each addition, the signal intensity was recorded once a stable value was reached. For the $10^6$ ppm data point, the reactor was bubbled with hydrogen (Figure 8).

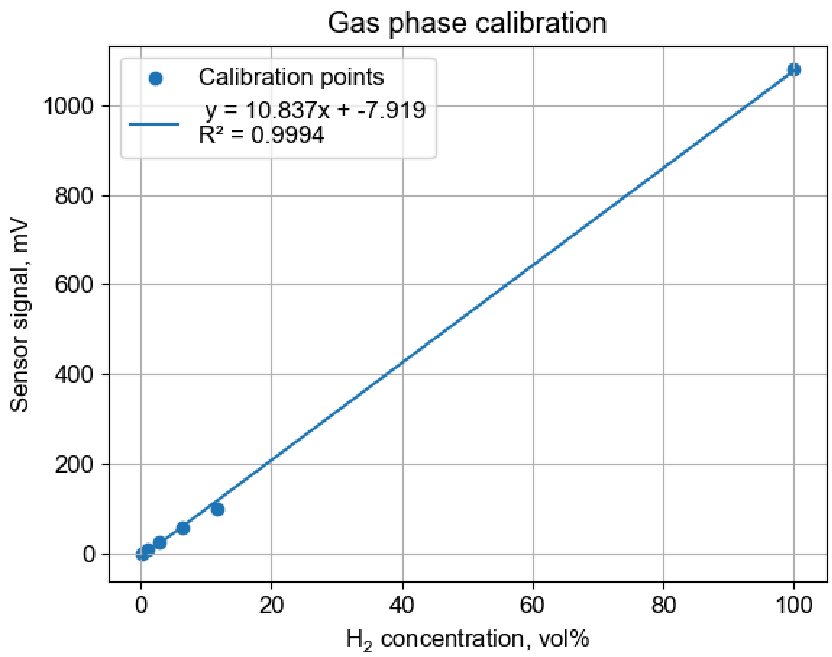


Figure 8: Calibration curve for Unisense sensor for $H_2$ measurements in gas phase

Both liquid- and gas-phase measurements demonstrate a linear sensor response over the investigated concentration range. Consequently, a two-point calibration was considered sufficient for routine daily calibration. This calibration was performed using 0 µM or 0 vol.% hydrogen as the lower calibration point and hydrogen-saturated water or water-saturated hydrogen as the upper calibration point for liquid- and gas-phase measurements, respectively.

The $O_2$ sensor was calibrated according to the manufacturer's instructions using a two-point calibration. The lower calibration point was obtained under fully degassed conditions, while the upper calibration point was taken under ambient conditions, corresponding to 21 vol.% $O_2$ in the gas phase and 256 µmol $L^{-1}$ $O_2$ in the liquid phase. The phase-shift difference (Δφ, dphi) values for gas- and liquid-phase measurements are summarized in table 5.

Table 5: Phase shift difference for $O_2$ sensor

| Gas phase calibration | | Liquid phase calibration | |
|---|---|---|---|
| dphi, ° (0 %) | dphi, ° (21 % $O_2$) | dphi, ° (0 µmol/L) | dphi, ° (256 µmol/L) |
| 7.05 | 49.76 | 6.97 | 49.32 |

### 4.4.2. Potential error sources for $H_2$ and $O_2$ measurements

Both the $H_2$ and $O_2$ measurements are subject to several potential sources of error. First, both sensors require calibration, which constitutes an inherent source of uncertainty. This error was minimized by performing the calibration carefully according to the manufacturer's instructions, with extended gas sparging periods. This approach reduces uncertainties

associated with incomplete equilibration. Details of the calibration procedure are provided in Section 4.4.1.

Another potential error source arises from the temperature dependence of both —$H_2$ and $O_2$— sensors. This error was minimized by using thermocouples positioned inside the reactor for temperature compensation, thereby largely eliminating temperature-related deviations. Nevertheless, the small nearly instantaneous temperature change (<0.5 °C) upon the onset of irradiation may introduce errors during the first seconds of irradiation. This consideration is —in part— the reason why the first 60 s of data after the start of irradiation were excluded from data evaluation.

The pressure increase during the reaction may also influence the measurements, particularly for gas-phase detection. However, under the applied experimental conditions below 1.5 vol.% of gas is formed in total during experiments involving gas-phase measurements. Assuming ideal gas behavior, this corresponds to a pressure increase of approx. 15 mbar. Such a small pressure change is negligible and is therefore not expected to significantly affect the measured gas concentrations or the derived reaction rates.

Stray light represents another potential error source for the optical $O_2$ sensor. However, stray light is detected by the sensor and internally corrected for during signal processing. Moreover, stray light would introduce a systematic error in the absolute $O_2$ concentration, which does not affect rate determination because rates are derived from relative changes in $O_2$ content rather than absolute concentrations.

For the $H_2$ sensor, electrical interference is a possible source of error, which may manifest as increased noise, spikes, or step-like features in the signal. These effects were minimized by avoiding interaction with the experimental setup during measurements. In cases where significant electrical interference was still observed, the corresponding datasets were discarded and the experiments repeated.

Finally, errors may arise from gas bubble formation at the sensor tips. Although this effect could not be completely prevented, each experiment was visually inspected after completion for bubble formation on either sensor. Measurements for which bubbles were observed were discarded and the experiments repeated.

### 4.4.3. Validation of $H_2$ gas phase measurements by gas chromatography

To validate the Unisense hydrogen sensor, the $H_2$ concentration was determined in a single setup using both the sensor and gas chromatography (GC).

Gas chromatographic measurements were performed using a Shimadzu Nexis GC-2030 equipped with a 5 Å molecular sieve column and helium as the carrier gas. The GC was calibrated using dry hydrogen with a four-point calibration (0.25, 0.50, 0.75, and 1.00 vol.% $H_2$).

For the validation experiments, a 5 mL GL14/NS14 Schlenk flask was used. The $H_2$ sensor was connected to the GL14 port using a BOLA fitting, while the NS14 port was sealed with a

septum. The PT1000 temperature sensor used for temperature compensation was placed adjacent to the flask in the water bath.

Three different hydrogen concentrations (0.05, 0.30, and 1.00 vol.% $H_2$) were investigated. Prior to addition, the hydrogen gas was bubbled through water to ensure water-saturated hydrogen.

Hydrogen concentrations were measured using both a Unisense hydrogen sensor and the gas chromatograph (Shimadzu Nexis GC-2030). For the validation, 0.05 vol.% $H_2$ was first added to the flask and the system was allowed to equilibrate until a constant sensor signal was obtained. Subsequently, a 100 µL gas sample was withdrawn through the septum and analyzed by GC. Next, an additional 0.25 vol.% $H_2$ was introduced, yielding a total concentration of 0.30 vol.% $H_2$, and the measurements were repeated using both methods. For the 1.00 vol.% data point, a further 0.70 vol.% $H_2$ was added.

The results obtained using both methods are summarized in table 6 and Figure 9.

Table 6: Comparison of the hydrogen concentrations determined Unisense $H_2$ sensor and gas chromatography

| **Theoretical $H_2$ content, vol.%** | **Hydrogen content determined with Unisense hydrogen sensor, vol.%** | **Hydrogen content determined with GC, vol.%** |
|---|---|---|
| 0.05 | 0.0517 | 0.252 |
| 0.30 | 0.3001 | 0.588 |
| 1.00 | 1.004 | 1.151 |

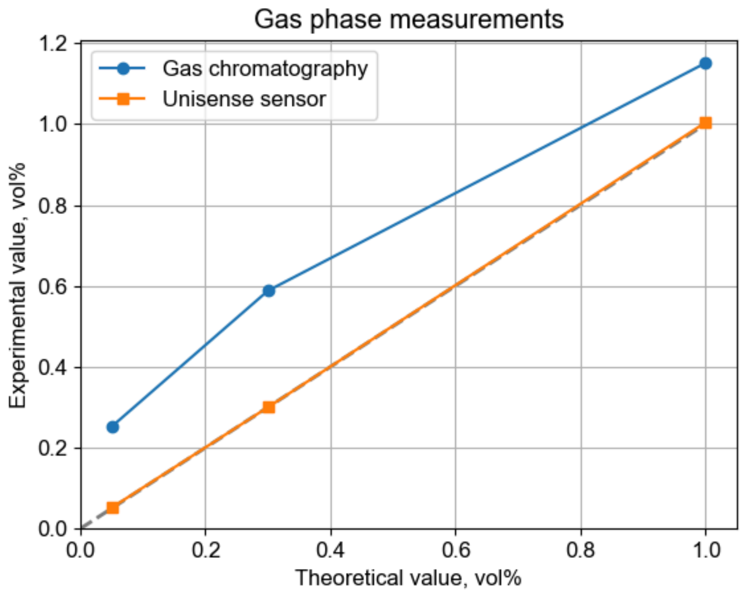


Figure 9: Comparison of the results obtained from the measurements with Unisense $H_2$ sensor and gas chromatography.

The values obtained with the Unisense hydrogen sensor are in excellent agreement with the theoretical concentrations (dashed line in the plot). In contrast, the GC measurements show

significant deviations from the theoretical values (with a relatively consistent offset across all datapoints, which can likely be attributed to the GC calibration).

# 5. Photocatalytic experiments with $O_2$ and $H_2$ simultaneous detection

## 5.1. Photocatalyst suspension preparation

In each photocatalytic test, the suspension with photocatalyst concentration 0.5 $mg.mL^{-1}$ was prepared. Al:$SrTiO_3$ loaded with $Rh_{2-y}Cr_yO_3$ (12.5 mg) was weighed in a 50 mL vial followed by addition of $H_2O$ (25 mL) (or $D_2O$). Next, the suspension was shaken on a vortex mixer for 3 min for homogeneous distribution of photocatalyst particles in the volume of the suspension. Afterwards, the vial was covered with Al foil before further use.

## 5.2. Main steps in photocatalytic tests

For each experiment, the reactor was placed in the double-walled beaker, the thermostat was set to the desired temperature, and filled with water for the $H_2$ sensor calibration procedure.

Next, the reactor was deassembled, degreased, washed with fresh portions of water and dried with acetone and compressed air.

Afterwards, the LED was attached to the irradiation chamber. The reactor was assembled again, PTFE stirring bar and the prepared photocatalyst suspension (25 mL, described in chapter 5.1) was transferred to the reactor using glass pipette followed by the addition of three sensors ($O_2$ sensor, PT100 and PT1000 sensors), sensor heights were adjusted, when needed, to be comparable. Degassing was performed for approx. 30 min using argon sparging with a PTFE cannula inserted *via* the valve outlet. Afterwards, the Unisense hydrogen sensor was introduced under argon counterstream. The needle tip was oriented toward the LED.

The main steps of the photocatalytic test are presented in Figure 10.

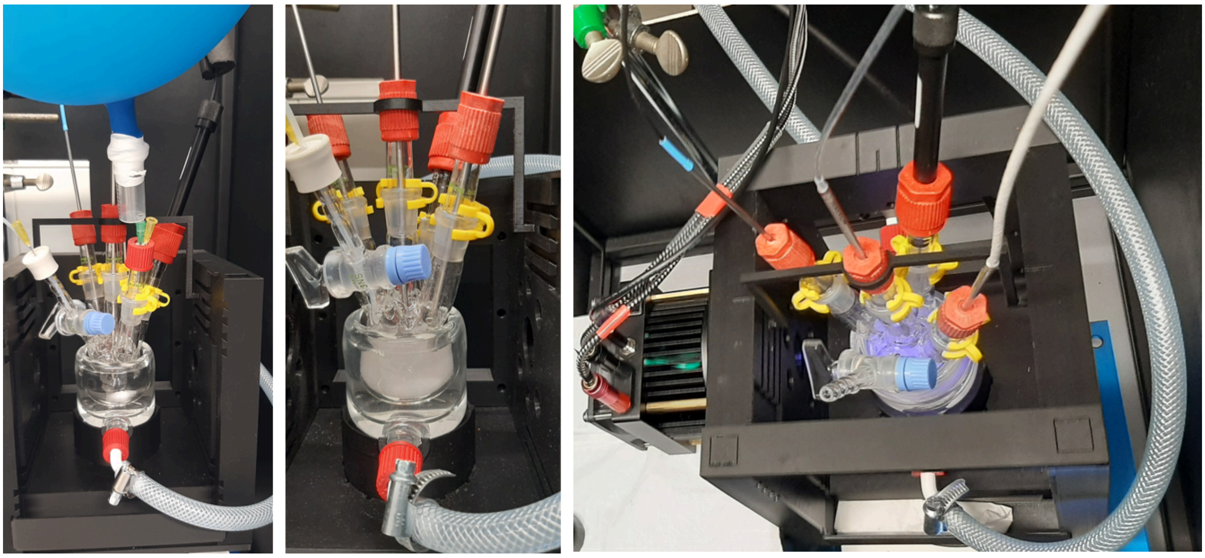

Figure 10: Important steps during photocatalytic tests: calibration of $H_2$ sensor under degassed conditions (left), degassing of the photocatalyst suspension (center), after start of irradiation, top view (right)

After sensor installation, the PTFE cannula was removed and the reactor was closed. A pre-reaction baseline was recorded for 10 min, followed by irradiation for 15 min and a post-reaction baseline for 10 min.

# 6. Overview of performed photocatalytic tests

Summary of the experimental conditions used in experiments is provided in table 7 below.

Table 7: Overview of main screening parameters

| **Parameters** | **Values** |
|---|---|
| Irradiance, mW·cm$^{-2}$ | 20, 50, 100, 150 |
| Temperature | 10, 20, 30 |
| Co-catalyst loading (Rh, Cr content, wt%) | 0.05, 0.1, 0.2 |
| Catalyst concentration, mg·mL$^{-1}$ | 0.5 |
| Dispersion medium | $H_2O$, $D_2O$ |

Groups of photocatalytic tests according to the varied conditions (irradiance, temperature, co-catalyst loading, dispersion medium, measurements in gas phase) were classified in the following manner (according to table 8) (for feasibility, the varied values in each group are highlighted with bold).

Table 8: Classification of performed photocatalytic tests

| **Group name** [a] | **Irradiance, $mW \cdot cm^{-2}$** | **Temperature, °C** | **Co-catalyst loading, Rh,Cr wt%** | **$D_2O$** | **Experiment number** |
|---|---|---|---|---|---|
| Reference | 50 | 20 | 0.1 | No | NB-316, NB-319, NB-320, NB-329, NB-331, NB-336, NB-339, NB-348, NB-353, NB-356 |
| Irradiance | **20** | 20 | 0.1 | No | NB-325, NB-326, NB-337, NB-345 |
| | **100** | 20 | 0.1 | No | NB-318, NB-322 |
| | **150** | 20 | 0.1 | No | NB-344, NB-347 |
| Temperature | 50 | **10** | 0.1 | No | NB-351, NB-359, NB-360 |
| | 50 | **30** | 0.1 | No | NB-330, NB-334 |
| Loading | 50 | 20 | **0.05** | No | NB-327, NB-332, NB-354, NB-357 |
| | 50 | 20 | **0.2** | No | NB-328 NB-333, NB-355, NB-358 |
| $D_2O$ | 50 | 20 | 0.1 | **Yes** | NB-346, NB-349 |
| Gas phase | 50 | 20 | 0.1 | No | NB-312, NB-361, NB-362, |

| | | | | | |
|---|---|---|---|---|---|
| | | | | | NB-363, NB-364 |
| Gas phase $D_2O$ | 50 | 20 | 0.1 | **Yes** | NB-365, NB-366, NB-367 |

[a] General reaction conditions: 25 mL $H_2O$, 0.5 mg (catalyst)·$mL^{-1}$, 365 nm LED irradiation, 10 min pre-reaction baseline, 15 min irradiation, 10 min post-reaction baseline

# 7. Analytical data

## 7.1. Spectrum of light source

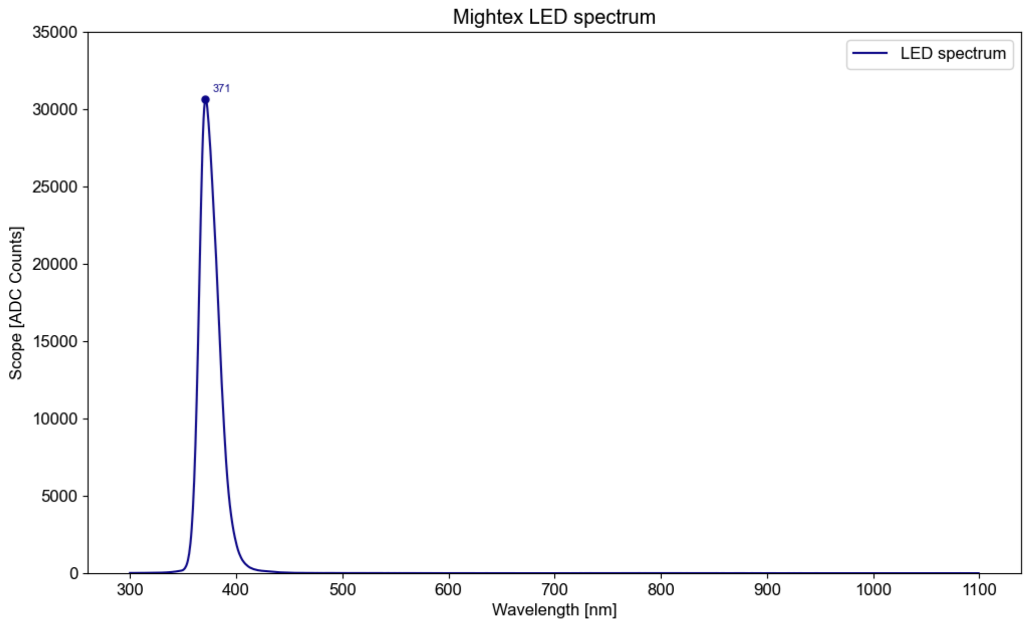


Figure 11: Emission spectrum of 365 nm LED.

# 8. Data processing and analysis

The entire code (Python) for data analysis and processing and analysis is open-source and can be found in the GitHub repository provided on the title page.

## 8.1. Detailed description of data processing workflow

From the raw experimental data the reaction phase is selected by selecting the interval between the start and end point of irradiation. Furthermore, the first 60 s after the start of

irradiation are removed due to diffusion of gases into the sensors causing an induction period.
The data is then shifted on the x-axis so that the start of irradiation is at $t$ = 0 s. Furthermore, the data is shifted on the y-axis so that the initial concentration is equal to 0 μmol.L$^{-1}$.
For gas phase data, the raw data (which is in the unit Pa for $H_2$ and vol% for $O_2$) is converted to μmol based on the volume of the gas phase, normal atmospheric pressure (101.325 Pa) and the ideal gas law. Furthermore, the amount of gases in μmol is scaled to the amount of gas that would be produced by one liter of liquid phase (using the experimental liquid phase volume), to obtain the unit μmol.L$^{-1}$ (for consistent units across liquid and gas phase).

To the thus obtained reaction data, a polynomial is fitted (4th order polynomial for liquid phase data, 3rd order polynomial for gas phase data). Furthermore, the experimental data is smoothed using a Savitzky-Golay filter (for $H_2$: window size: 30, polynomial order: 1; for $O_2$: window size: 10, polynomial order: 3). Both the fitted polynomial and the smoothed data are numerically differentiated to obtain the corresponding rate data. The maximum of the differentiated polynomial is picked to obtain the maximum rate.

## 8.2. Liquid/gas phase mass transport during photocatalytic water splitting

Using the photocatalytic reactor set-up and prepared photocatalyst, we initially wanted to investigate the mass transport of $H_2$ and $O_2$ from the liquid into the gas phase during photocatalytic water splitting. We performed two sets of experiments, where we detected $H_2/O_2$ once in the liquid and once in the gas phase, measuring the evolution of both gases over time.
In the liquid phase, immediate formation of both $H_2$ and $O_2$ can be observed (see Figure 12), although the rate is slightly slower at the beginning. In the gas phase, a pronounced induction period of around three minutes is observed due to diffusion of the gases from the liquid to the gas phase. Experimentally, it can be seen that the ratio of $H_2$ and $O_2$ is not constant over time: in the liquid phase, it starts at ca. 2:1 $H_2/O_2$ but quickly decreases to a ratio of 1.4:1. In the gas phase, the initial ratio is around 2.5:1 and gradually decreases to 2:1.

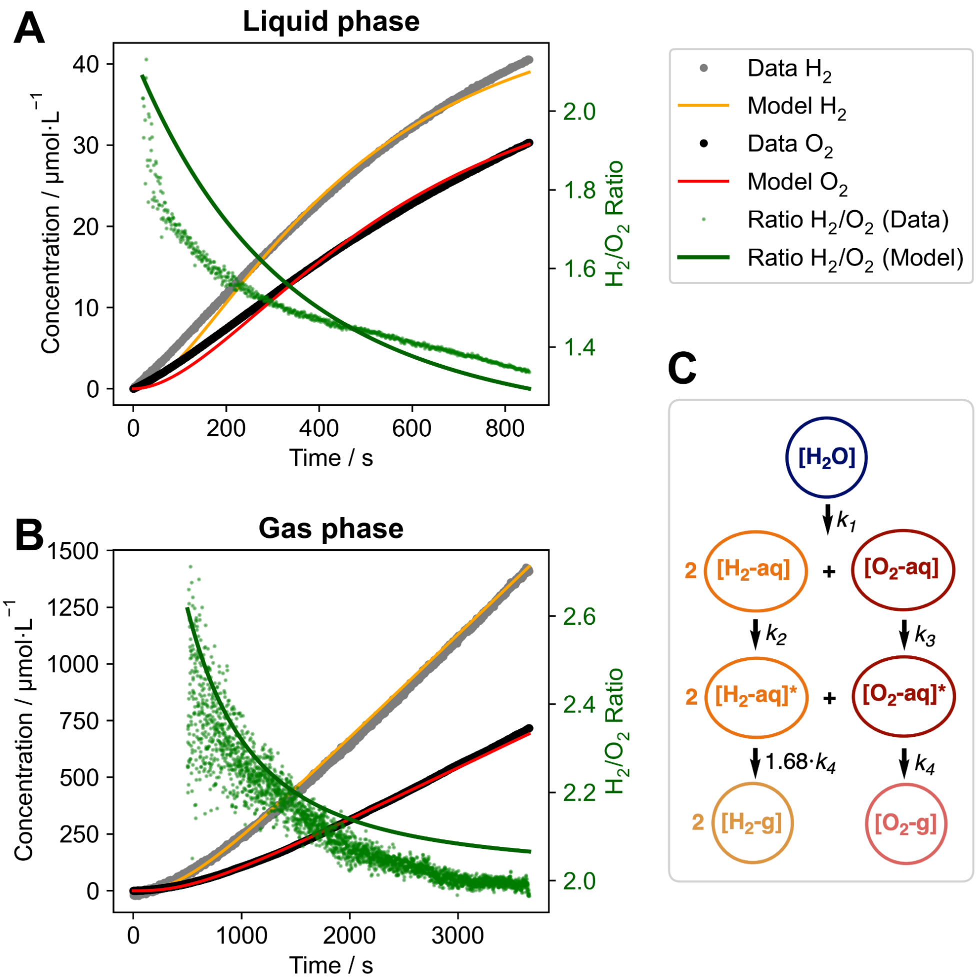


Figure 12: Experimental data and kinetic modelling of $H_2/O_2$ evolution in the liquid (A) and gas phase (B). For both measurements the evolution of the gases over time is shown (black and grey dots) as well as the ratio between the two gases (right axis, green dots). The concentration of the liquid phase gases is given in µmol.L$^{-1}$ and the gas phase concentration is given in an analogous unit of µmol.L$^{-1}$, which indicates the amount of gases formed per liter of irradiated liquid phase volume (to have consistent units for the kinetic modelling). To both the liquid and the gas phase data one kinetic model (C) with one set of rate constants is fitted (black, grey and green lines). The optimized values for the rate constants are: $k_1$ = 4.1.10$^{-9}$ s$^{-1}$, $k_2$ = 2.3.10$^{-3}$ s$^{-1}$, $k_3$ = 2.2.10$^{-3}$ s$^{-1}$, $k_4$ = 5.8.10$^{-3}$.

To rationalize these observations, we fitted to the data a simple kinetic model (see Figure 12 C). This model is deliberately chosen to be as simple as possible and is only intended to provide a phenomenological description of the experimental observations.

The model is composed of the following reaction steps:

Step 1: [$H_2O$] > 2 [$H_2$-aq] + [$O_2$-aq], $k_1$
Step 2: [$H_2$-aq] > [$H_2$-aq]*, $k_2$
Step 3: [$O_2$-aq] > [$O_2$-aq]*, $k_3$
Step 4: [$O_2$-aq]* > [$O_2$-g], $k_4$
Step 5: [$H_2$-aq]* > [$H_2$-g], $k_4$ · 1.68

Step 1 described the water splitting reaction forming two equivalents of $H_2$ and one equivalent of $O_2$. Steps 2 and 3 describe the diffusion of $H_2$ and $O_2$ within the liquid phase, respectively, especially to reach the sensors. Steps 4 and 5 describe the diffusion of both gases from the liquid to the gas phase. Here, the same rate constant ($k_4$) is used for both cases, but for $H_2$ it is multiplied by a factor of 1.68, which is the ratio between the solubilities/Henry's constants of the two gases.

Fitting this model simultaneously to the $H_2$ and $O_2$ data in the liquid and gas phase (with just one set of values for the rate constants, using a differential evolution algorithm) gives acceptable agreement with both the time evolution of the gases as well as the observed ratios over time (see Figure 12).

The evolution of the $H_2/O_2$ ratios over time can be understood based on the different solubilities of $H_2$ and $O_2$ in water (as described by the ratio of the Henry's law constants): due to the lower solubility of $H_2$, it diffuses out of the liquid phase more quickly, leading to a lower $H_2/O_2$ ratio in the liquid phase (converging to roughly 1.2:1) and correspondingly a higher initial $H_2/O_2$ ratio in the gas phase. In the gas phase, the $H_2/O_2$ ratio does converge to the expected 2:1 ratio over time.

It should be stated clearly that this simplified model is purely phenomenological and is only intended to provide a qualitative understanding for the reason why the $H_2/O_2$ ratios in the liquid and gas phase evolve over time.

## 8.3. Processed experimental data

Liquid phase data:

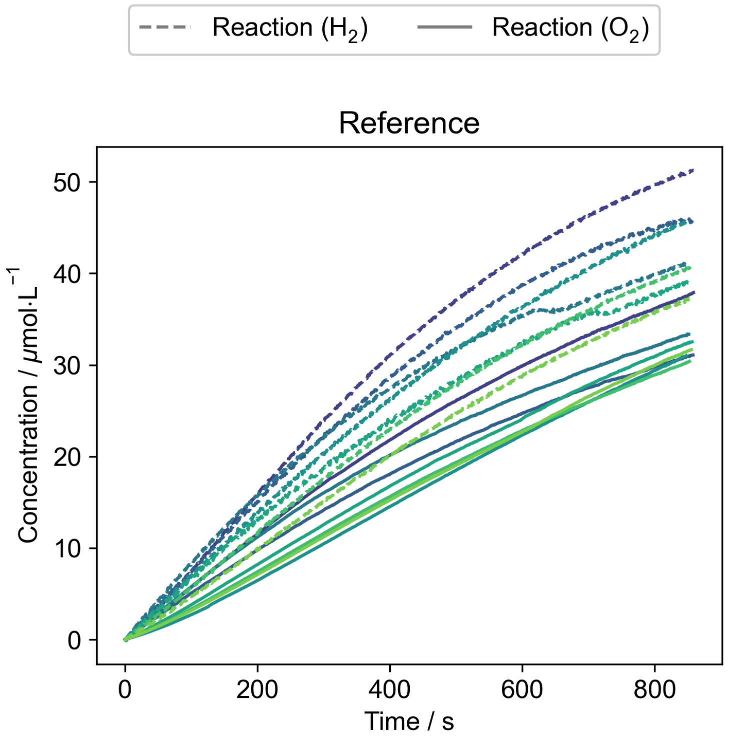


Figure 13: Experimental data for $H_2/O_2$ simultaneous measurements (reference conditions)

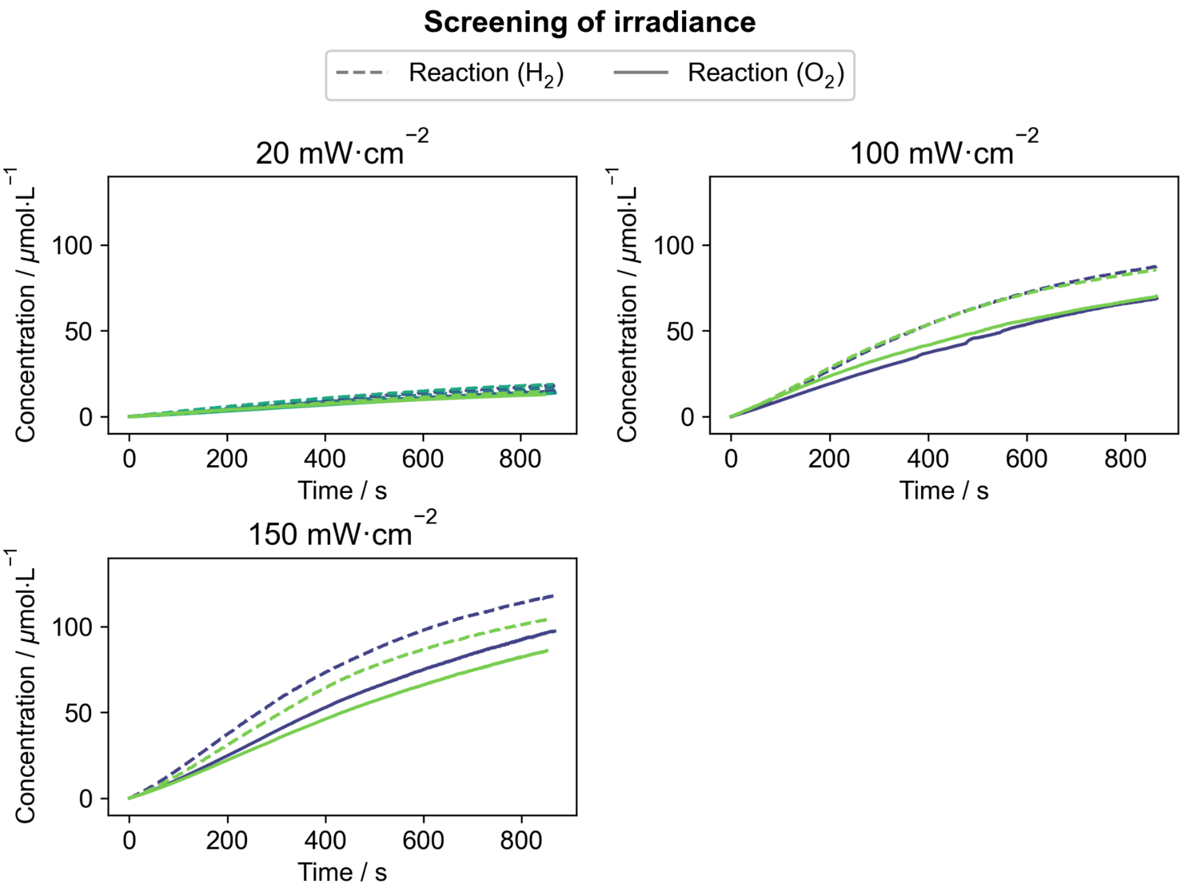


Figure 14: Experimental data for $H_2$/$O_2$ simultaneous measurements (screening of irradiance: 20 mW.cm$^{-2}$, 100 mW.cm$^{-2}$, 150 mW.cm$^{-2}$)

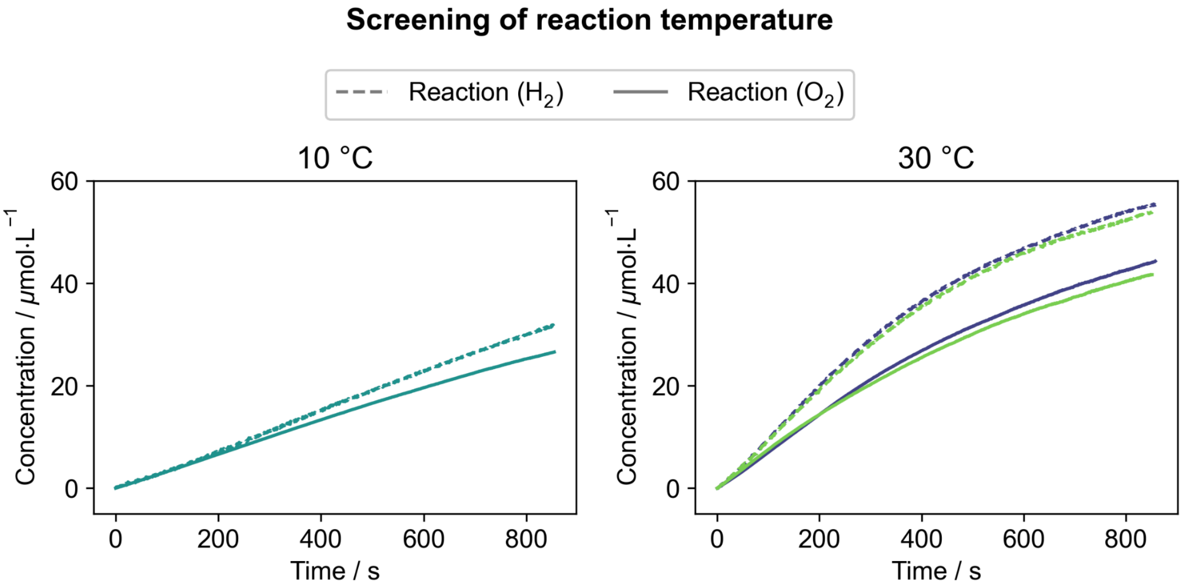


Figure 15: Experimental data for $H_2$/$O_2$ simultaneous measurements (screening of temperature: 10 °C, 30 °C)

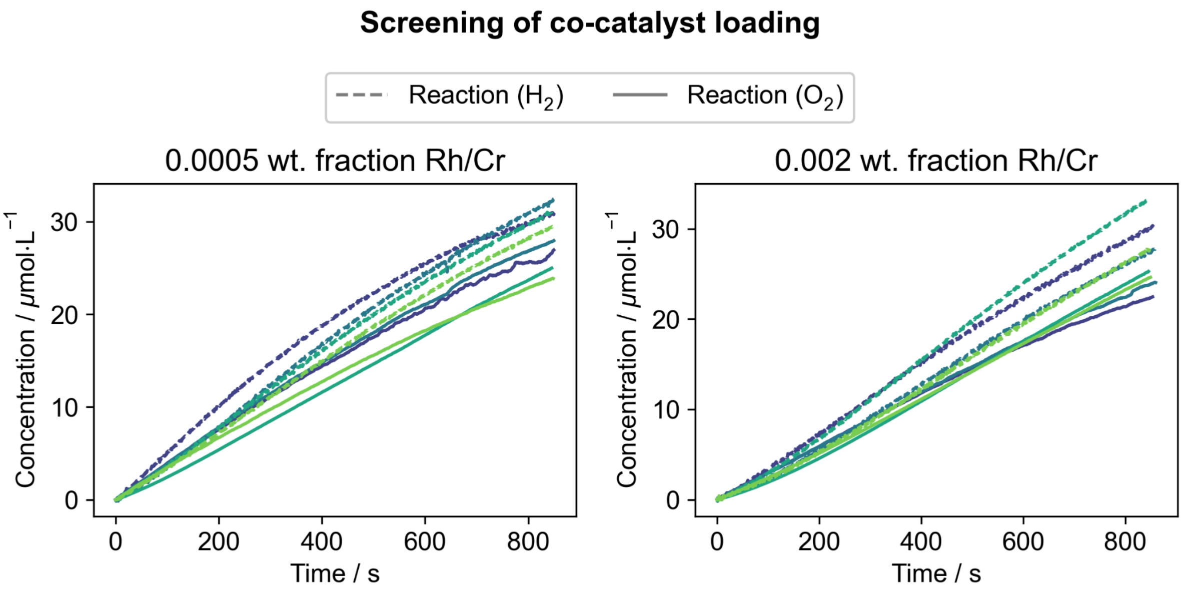


Figure 16: Experimental data for $H_2/O_2$ simultaneous measurements (screening of co-catalyst loading: 0.0005 wt. fraction Rh/Cr, 0.002 wt. fraction Rh/Cr)

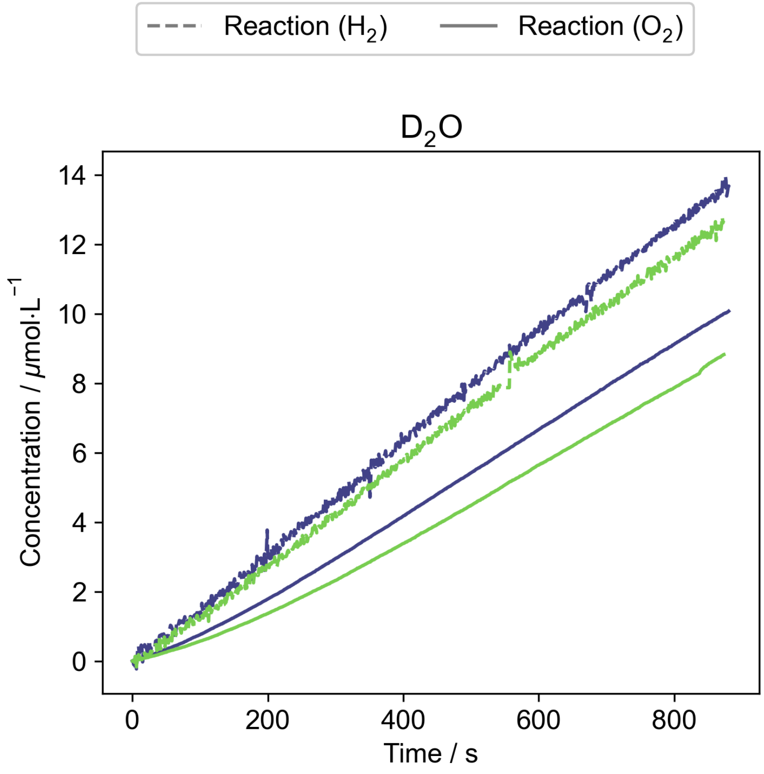


Figure 17: Experimental data for $H_2/O_2$ simultaneous measurements (kinetic isotope effect investigation)

Gas phase data:

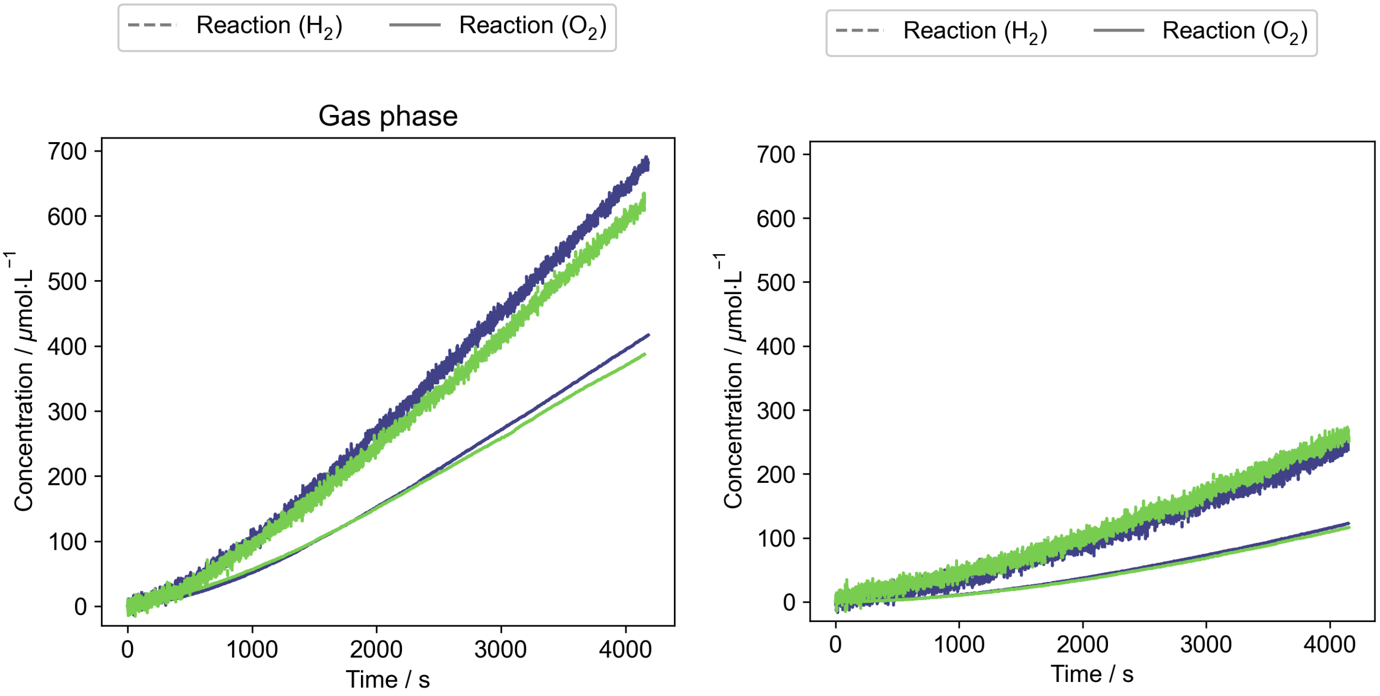


Figure 18: Experimental data for $H_2$/$O_2$ simultaneous measurements in gas phase (left: $H_2O$ as a dispersion medium, right: $D_2O$ as a dispersion medium for kinetic isotope effect investigation)

## 8.4. Arrhenius analysis of temperature dependent data

The temperature-dependent liquid phase data was analyzed based on Arrhenius' equation, through standard logarithmic linearization, followed by linear regression to determine the activation energy based on both the $H_2$ and $O_2$ data:

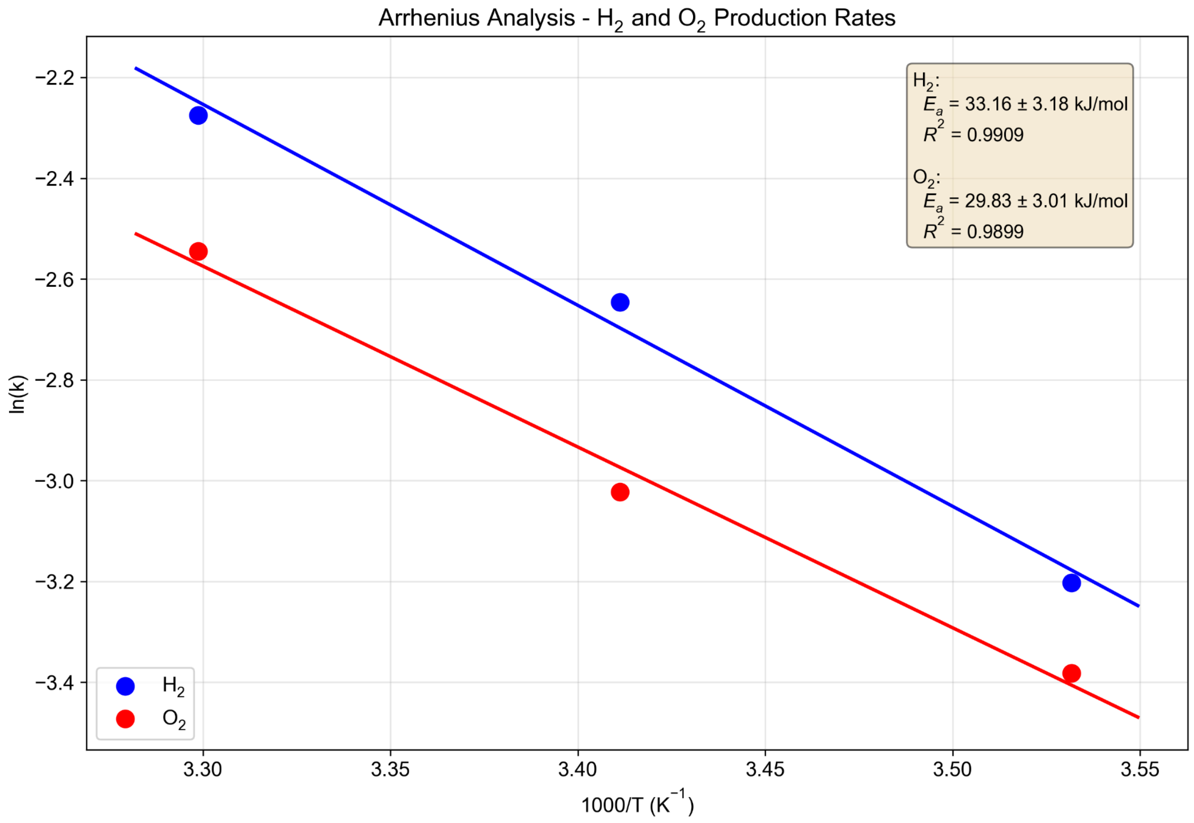


Figure 19: Arrhenius analysis of temperature dependent liquid phase experimental data to determine the thermal activation energy

## 8.5. Overview of kinetic results

Numerical values for the kinetic results (provided in Figure 3 of the manuscript) are provided in table 9 below.

Table 9: Maximum rates of $H_2$ and $O_2$ formation in performed photocatalytic tests

| **Group name** | **Irradiance, mW·cm$^{-2}$** | **Temperature, °C** | **Co-catalyst loading, RhCr wt%** | **$D_2O$** | **Max rate, µmol·L$^{-1}$·s$^{-1}$** | | |
|---|---|---|---|---|---|---|---|
| | | | | | **Min value** | **Max value** | **Mean value** |
| Reference | 50 | 20 | 0.1 | No | $O_2$: 0.041<br>$H_2$: 0.052 | $O_2$: 0.062<br>$H_2$: 0.083 | $O_2$: 0.049<br>$H_2$: 0.071 |

| | | | | | | | |
|---|---|---|---|---|---|---|---|
| Irradiance | **20** | 20 | 0.1 | No | $O_2$: 0.018<br>$H_2$: 0.021 | $O_2$: 0.022<br>$H_2$: 0.032 | $O_2$: 0.020<br>$H_2$: 0.025 |
| | **100** | 20 | 0.1 | No | $O_2$: 0.104<br>$H_2$: 0.145 | $O_2$: 0.135<br>$H_2$: 0.148 | $O_2$: 0.119<br>$H_2$: 0.146 |
| | **150** | 20 | 0.1 | No | $O_2$: 0.123<br>$H_2$: 0.178 | $O_2$: 0.144<br>$H_2$: 0.200 | $O_2$: 0.133<br>$H_2$: 0.189 |
| Temperature | 50 | **10** | 0.1 | No | $O_2$: 0.034<br>$H_2$: 0.041 | $O_2$: 0.034<br>$H_2$: 0.041 | $O_2$: 0.034<br>$H_2$: 0.041 |
| | 50 | **30** | 0.1 | No | $O_2$: 0.076<br>$H_2$: 0.100 | $O_2$: 0.081<br>$H_2$: 0.106 | $O_2$: 0.078<br>$H_2$: 0.103 |
| Loading | 50 | 20 | **0.05** | No | $O_2$: 0.031<br>$H_2$: 0.039 | $O_2$: 0.044<br>$H_2$: 0.054 | $O_2$: 0.038<br>$H_2$: 0.045 |
| | 50 | 20 | **0.2** | No | $O_2$: 0.030<br>$H_2$: 0.037 | $O_2$: 0.033<br>$H_2$: 0.044 | $O_2$: 0.031<br>$H_2$: 0.039 |

| | | | | | | | |
|---|---|---|---|---|---|---|---|
| Gas phase | 50 | 20 | 0.1 | No | $O_2$: 0.113 $H_2$: 0.179 | $O_2$: 0.124 $H_2$: 0.194 | $O_2$: 0.119 $H_2$: 0.186 |
| Liquid phase $D_2O$ | 50 | 20 | 0.1 | **Yes** | $O_2$: 0.012 $H_2$: 0.016 | $O_2$: 0.013 $H_2$: 0.017 | $O_2$: 0.012 $H_2$: 0.016 |
| Gas phase $D_2O$ | 50 | 20 | 0.1 | **Yes** | $O_2$: 0.044 $H_2$: 0.076 | $O_2$: 0.044 $H_2$: 0.084 | $O_2$: 0.044 $H_2$: 0.080 |

# 9. References


1. Z. Zhao, R. V. Goncalves, S. K. Barman, E. J. Willard, E. Byle, R. Perry, Z. Wu, M. N. Huda, A. J. Moulé, F. E. Osterloh, *Energy Environ. Sci.* **2019**,*12*, 1385-1395.
2. JCPDS-International Centre for Diffraction Data, Card No. 35-0734.
3. Y.-G. Lee, Y.-C. Cheng, Y.-T. Lin, J. C. S. Wu, W.-Y. Yu, M. M. Kržmanc, S. Gupta, E. Kotomin, *J. Phys. Chem. C* **2023**, 127 (21), 9981–9991.
4. D. Kowalczyk, P. Li, A. Abbas, J. Eichhorn, P. Buday, M. Heiland, A. Pannwitz, F. H. Schacher, W. Weigand, C. Streb, D. Ziegenbalg, *ChemPhotoChem.* **2022**, *6 (7)*, e202200044.
